# Supplementary material for: Raised Leptin and Pappalysin2 cell-free RNAs are the hallmarks of pregnancies complicated by preeclampsia with fetal growth restriction
Source: Nat Commun. 2025 Jul 18;16:6614. doi: 10.1038/s41467-025-61931-7 (PMC12274584; doi:10.1038/s41467-025-61931-7)
Supplement: Supplementary file 1 — Supplementary Information [file 41467_2025_61931_MOESM1_ESM.pdf]

Supplementary Information

Selection of RNA-seq quantification method

We assessed several RNA-seq data processing pipelines and selected the most accurate approach based on the performance of predicting fetal sex by measuring the extent of chromosome Y (chrY) gene expression level. Y chromosome transcripts are lowly expressed and therefore likely to be challenging to quantify in maternal plasma. This task, however, is further complicated because 1) there are multiple copies of chrY gene families, such as TSPY which contains ~30 copies, and 2) most chrY genes have close homologues in chromosome X – some chrY genes show ~99% identity with their corresponding X-linked homologues. Thus, evaluating quantitation methods based on fetal sex determination uses real data (not synthetic data) with highly related transcripts that are poorly expressed. The study from Godfrey et al. *(54)* showed that *kallisto (55)* performed better than the two most used conventional methods (*TopHat2 (56)*+ *featureCounts (57)* and *TopHat2 (56)* + *Cufflinks(58)*) in terms of quantifying the expression level of male specific chrY genes precisely. Recently, Srivastava et al. *(59)* showed improved performance of their new methodology, so called selective alignment (SA) implemented in the *salmon* software package. This outperformed various existing methods, including *kallisto*, in both simulated and real RNA-seq datasets. Therefore, we tested the performance of *salmon* (v1.5.2) *(46)* with the following two approaches: 1) the mapping-based mode, and 2) the alignment-based mode. For the mapping-based mode, we applied so-called selective alignment option and employed "decoy" sequences – this approach was named as "Salmon (SA mode)". For the alignment-based mode, we used *HISAT2* (v2.2.1) *(60)*, a successor of TopHat2 *(56)*, followed by salmon in the alignment mode – this approach was named as "HiSat2+Salmon". Firstly, we selected 100 RNA-seq samples randomly and down-sampled the original sequencing reads to 1, 5 and 10 million per sample (**Supplementary Table 1**) using *seqtk* (v1.2-r101-dirty) *(61)*. We ran the two pipelines for each set of down-sampled reads, then measured the read counts for protein-coding chrY genes – there were 42 such chrY genes based on the Ensembl annotation v88 *(48)*. If there was at least one read quantified in any of the 42 chrY genes, such a sample was simply predicted as male; otherwise, female. Then we tabulated confusion matrices by the numbers of true-positives (i.e. predicted as male and the true fetal sex being male; TP), true-negatives (i.e. predicted as female as the true fetal sex being female; TN), false-positives (i.e. predicted as male but the true fetal sex being female; FP), and false-negative (i.e. predicted as female but the true fetal sex being male; FN). We calculated the precision (also known as, positive predictive value), recall (also known as, true positive rate or sensitivity), and the F_1 score as the followings:

$$Precision = TP/(TP + FP),$$

$$Recall = TP/(TP + FN),$$

$$F_1\ score = 2\ \frac{Precision \times Recall}{Precision + Recall}$$

Finally, we chose the pipeline based on the higher value of F_1 score which is in favour of a smaller number of FP and FN.

For the 42 chrY genes, we found that a smaller number of reads was quantified from the female samples when "Salmon (SA mode)" was used compared to "HiSat2+Salmon" – this pattern was more noticeable from the samples of advanced gestation (**Supplementary Figure 1A**). Therefore, the number of FP and FN were smaller for "Salmon (SA mode)" (**Supplementary Table 2**), and the F1 score was higher for "Salmon (SA mode)" than "HiSat2+Salmon" (**Supplementary Figure 1B** and **Supplementary Table 2**). Based on the 10M down-sampled RNA-seq samples, there were 24 and 16 chrY genes quantified as having at least one read from the 100 randomly selected samples, by "HiSat2+Salmon" and "Salmon (SA mode)", respectively (**Supplementary Figure 1C**).

Assessing the effect of trimming the adaptor sequences

Having compared different approaches of using RNA-seq quantification methods as described above, we investigated the effect of trimming or no-trimming the adaptor sequences on the performance of predicting fetal sex. For this purpose, we used the 10 million down-sampled reads from the 100 randomly selected samples and ran *cutadapt* (v2.7 with Python 3.6.8) *(62)* with the following parameters:

-j 32 -a AGATCGGAAGAGCACACGTCTGAACTCCAGTCAC -A AGATCGGAAGAGCGTCGTGTAGGGAAAGAGTGTAGATCTCGGTGGTCGCCGTATCATT -q 20 -O 8 -m 20

We employed "Salmon (SA mode)" approach for both trimmed and un-trimmed input reads, then compared the number of read quantified on the 42 chrY genes, and the number of false classifications of fetal sex. Again, a sample is predicted as male if at least one read was quantified on any of the 42 protein-coding chrY genes as described before. We found that, regardless of trimming or not, the number of TP, TN, FP, and FN remained the same and the sum of read quantified on the 42 chrY genes also remained very similar ($R=0.999$, $P<2.2\times10^{-16}$, Pearson's correlation test with two-sided, **Supplementary Figure 1D**), even though there were two female samples with a slightly higher sum of chrY reads when quantified using trimmed input reads (one 12wkGA sample with a total of 1.887 reads from non-trimmed input and 2.886 from trimmed input; one 20wkGA sample with a total of 0.918 read from non-trimmed input and 0.919 read from trimmed input).

In conclusion, we employed “Salmon (SA mode)” without trimming the input raw sequencing reads.

Supplementary Table 1. The number of randomly selected samples by the gestational age group and the fetal sex.

		Fetal sex		
		**Female**	**Male**	**Sum**
**Gestational Age**	**12wk**	17	5	**22**
	**20wk**	18	7	**25**
	**28wk**	19	15	**34**
	**36wk**	11	8	**19**
	**Sum**	**65**	**35**	**100**

Supplementary Table 2. The confusion matrices of predicting fetal sex based on the down-sampled reads.

Down-sampled size	Measure	HiSat2+Salmon	Salmon (SA mode)
**1 million (1M)**	**TP**	25	22
	**TN**	62	65
	**FP**	3	0
	**FN**	10	13
	**F_1 score**	0.794	0.772
	**Precision**	0.893	1.000
	**Recall**	0.714	0.629
**5 million (5M)**	**TP**	31	29
	**TN**	46	62
	**FP**	19	3
	**FN**	4	6
	**F_1 score**	0.729	0.866
	**Precision**	0.620	0.906
	**Recall**	0.886	0.829
**10 million (10M)**	**TP**	32	31
	**TN**	37	58
	**FP**	28	7
	**FN**	3	4
	**F_1 score**	0.674	0.849
	**Precision**	0.533	0.816
	**Recall**	0.914	0.886

TP: True Positive, TN: True Negative, FP: False Positive, FN: False Negative

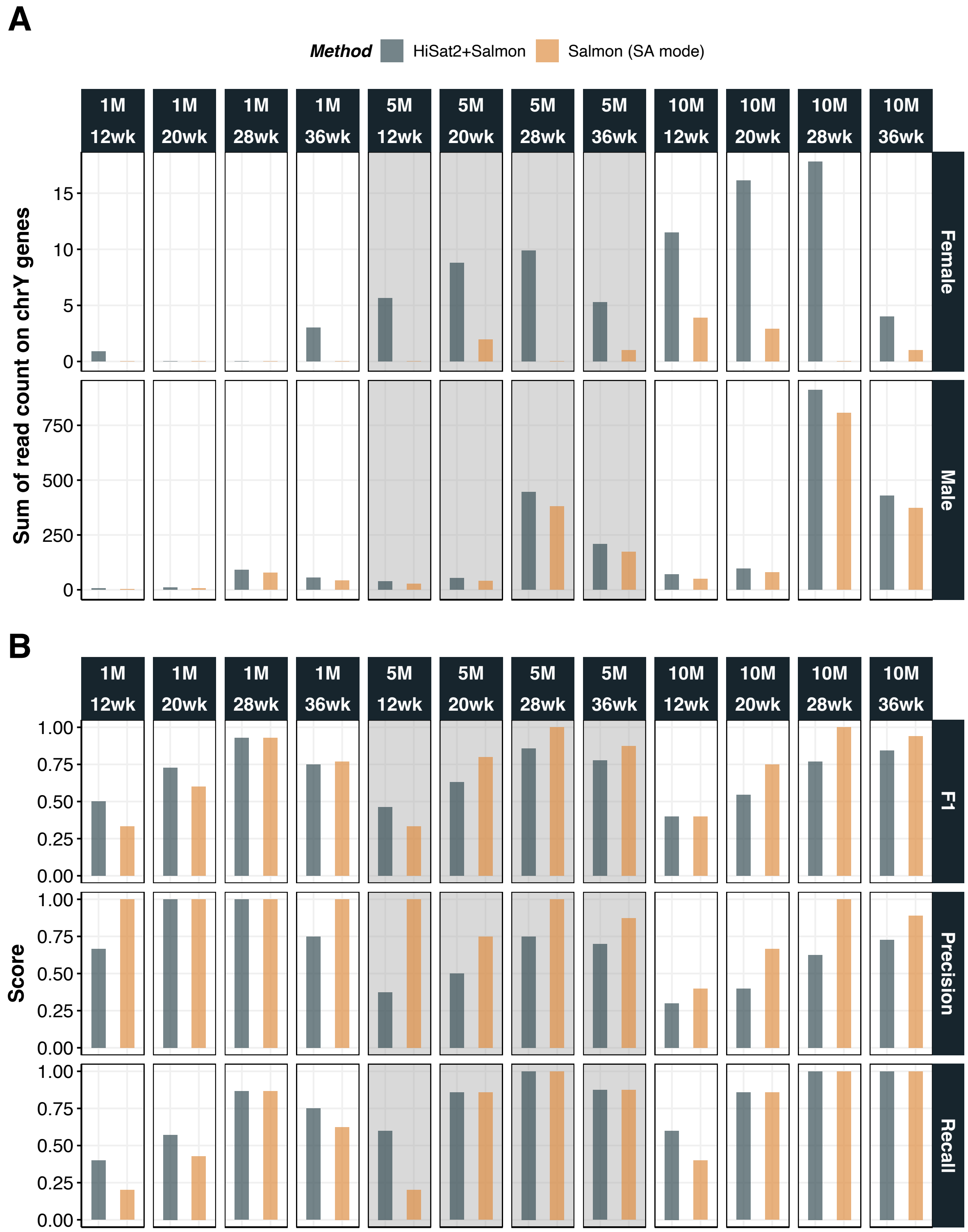

Supplementary Figures 1A and 1B. The sum of reads across the 42 chrY genes measured by two methods and their performance of predicting the fetal sex. From the 100 randomly selected RNA-seq samples, as shown in **Supplementary Text Table 1**, their original raw reads were down-sampled to 1 million (1M), 5 million (5M), and 10 million (10M), and the level of gene expression was quantified by two methods: 1) HiSat2+Salmon colored in grey, and Salmon (SA mode) colored in amber. (**A**) the sum of reads across the 42 protein-coding chrY genes are shown on the y-axis by the fetal sex and the four gestational ages groups (12wk, 20wk, 28wk and 36wk) per

down-sampled dataset (1M, 5M and 10M). **B**, the performance of predicting the fetal sex based on the existence of chrY signals (i.e. predicting male as being positive) is plotted using the precision (Precision), recall (Recall) and the F1-score (F1) which is the harmonic mean of the precision and recall. A sample is predicted as male if there was at least one read quantified in any of the 42 chrY genes. (A-B), the background color of 5M dataset is colored in grey to distinguish it from the 1M and 5M down-sampled datasets.

C

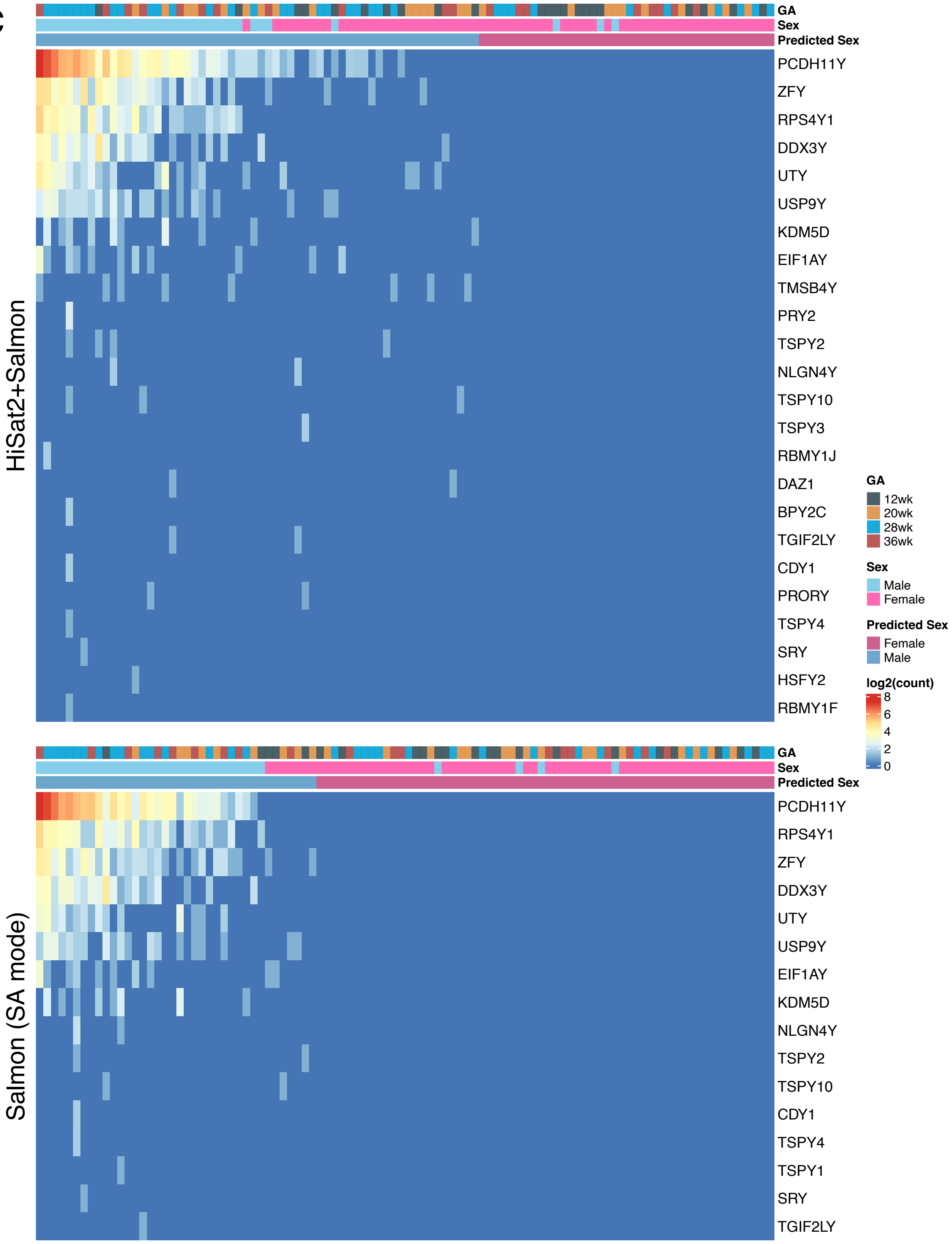

Supplementary Figure 1C. The Heatmaps of chrY genes quantified by the two methods in comparison. The read count quantified by "HiSat2+Salmon" and "Salmon (SA mode)" were used in the top and the bottom heatmap, respectively, based on the 10 million down-sampled reads from the 100 randomly selected RNA-seq samples, as shown in **Supplementary Table 1**, which are represented in the column (without label). Each row represents one of the 42 chrY protein-coding genes quantified as having at least one read in any of the 100 samples, and it is sorted by the sum across the 100 samples in the descending order. Likewise, the samples (i.e. columns) are sorted by the sum across the corresponding chrY genes (rows) in the descending order from the left (higher) to right (lower). There were 24 such chrY genes when quantified by "HiSat2+Salmon", and there were 16 such chrY genes when quantified by "Salmon (SA mode)". On top of each heatmap, samples are labeled in various colors for their gestational age (GA) and the real fetal sex (Sex), and the predicted sex (Predicted Sex). A sample is predicted as male if there was at least one read quantified in any of the 42 chrY genes. To avoid infinite value in log2 scale, a pseudo-count 1 was added to each cell.

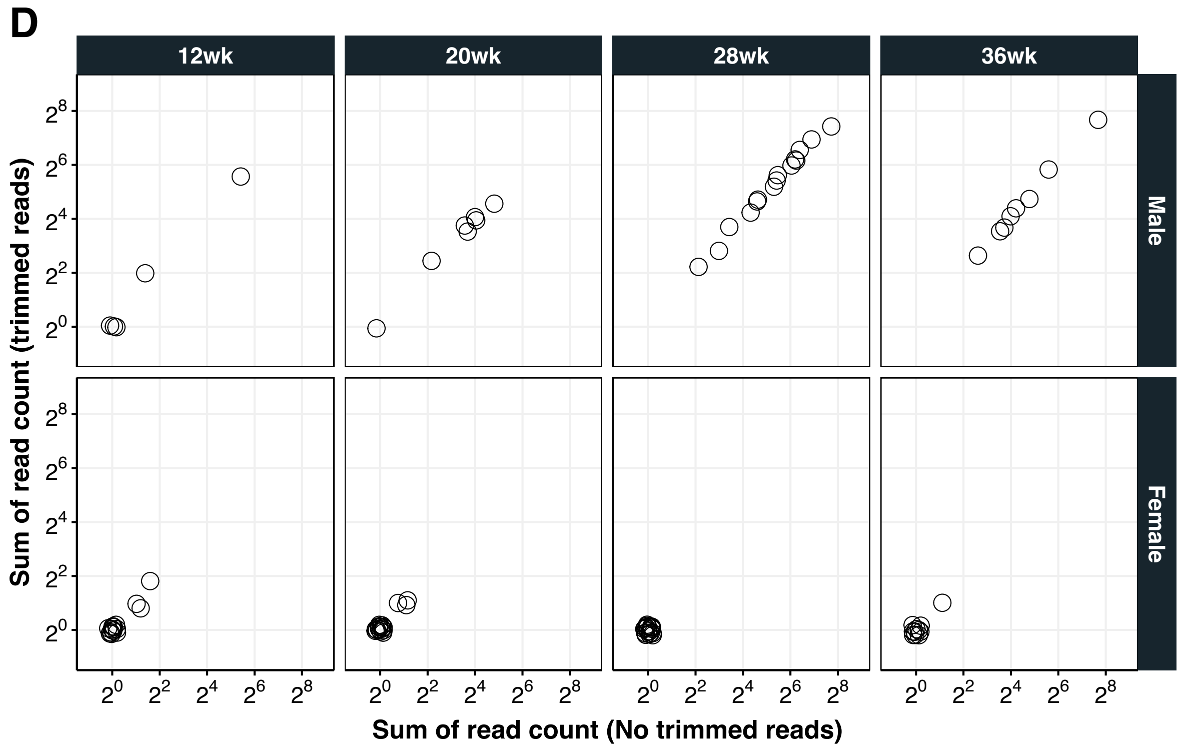

Supplementary Figure 1D. The sum of read count mapped to the 42 protein-coding chromosome Y genes with or without trimming reads. Each dot represents one of 100 randomly selected samples as shown in **Supplementary Table 1**. To avoid plotting the circles on the same position, a small random variation was added. Both the x-axis and y-axis represent the sum of total reads mapped across the 42 chrY protein-coding genes by using non-trimmed (x-axis) and trimmed (y-axis) raw reads. For both approaches, "Salmon (SA mode)" was used for quantification based on the 10 million down-sampled reads. In the top 4 panels (Male), the numbers of samples having zero read (therefore predicted as female shown in **Supplementary Figure 1C** – Salmon (SA mode)) are as the following: 3 (12wk), and 1 (20wk). In the bottom 4 panels (Female), the numbers of samples having at least one read (therefore predicted as male shown in **Supplementary Figure 1C** – Salmon (SA model)) are as the following: 3 (12wk), 3 (20wk), 1 (36wk).

A like-for-like comparison of validated AUCs using the combined model and the original model

Here we present the predictive performance of the 2- to 10-cfRNA models trained in the 28wkGA samples from the combined dataset from discovery and validation cohort – we called these models as "combined models" or preterm+term models because discovery cohort is from the preterm delivery and the validation cohort from the term delivery. Then, we compare their predictive performances to those from the 2- to 10-cfRNA models trained in the 28wkGA samples of discovery cohort – we call these models as the original models (or preterm models) as mentioned in the main text of the paper (see **Figure 4A**). The 2- to 10-cfRNAs in the combined and the original models were chosen by Elastic net. **Supplementary Data 9** shows the full comparison cross-validated AUCs using our POPS dataset and the external dataset from Munchel *et al.* In general, the combined models performed better than the original models when the performance was measured in validation cohort (term delivery), whereas the original model performed better than the combined model in discovery cohort (preterm delivery). In our internal validation, however, samples from the same participants were used in the combined models, so they are not truly independent, unlike the Munchel data set.

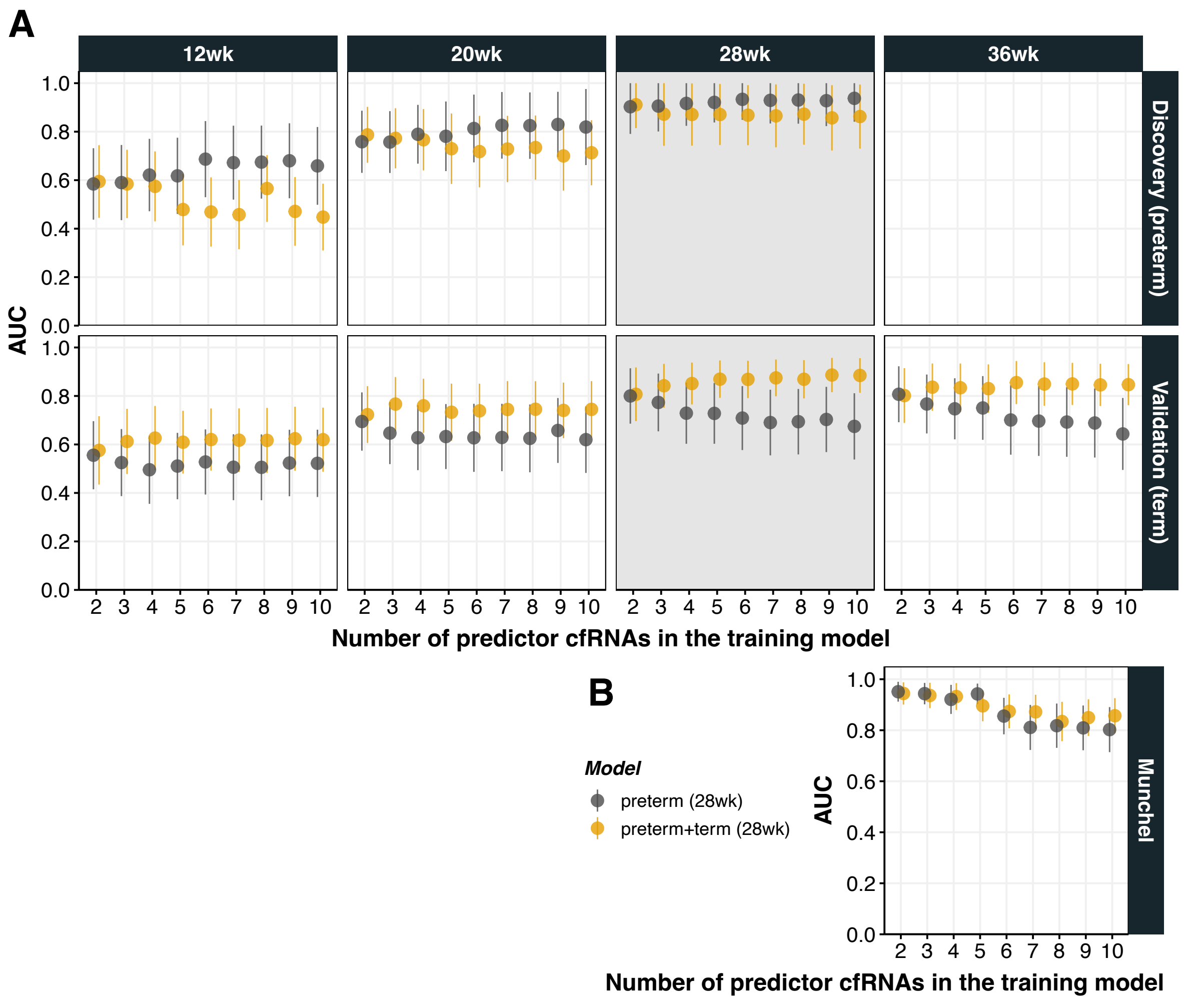

Supplementary Figure 2. A like-for-like comparison of the predictive performance between the original model and the combined model. The predictive performances (measured in AUC) are colored in black for the original model and colored in yellow for the combined model, separately in POPS internal dataset (**A**) and the external dataset from Munchel et al. (**B**). The panels colored in grey background in (**A**) indicate the training dataset (i.e., the 28wkGA samples) were used as a whole or partly when fitting the original and the combined models, respectively. Therefore, the AUC validated in discovery cohort is from the same dataset where the original models were trained, i.e. not cross validated. For both the original and the combined models, 2- to 10-cfRNAs were chosen by Elastic net in the corresponding dataset. See **Supplementary Data 9** for the source data for this figure. The mean AUC and its 95% confidence intervals are plotted.

The z-scores of LEP and PAPPA2 cfRNA

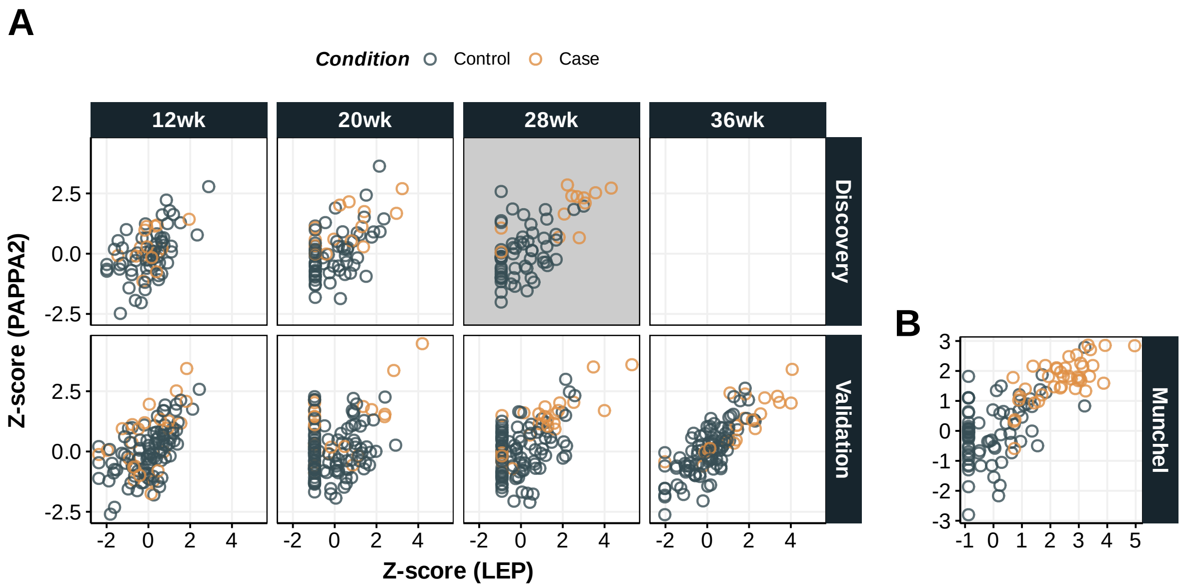

Supplementary Figure 3. The z-scores of LEP and LAPPA2 cfRNAs. The z-scores of LEP and PAPPA2 cfRNA are plotted by the gestational age in discovery and internal validation cohort (**A**), and in external validation dataset (**B**). In (A), the panel coloured in grey background indicates the training dataset (i.e., the 28wkGA samples of the discovery cohort) where the corresponding models were fitted. Note that the z-scores were transformed from the log2-scale of CPM (Count Per Million) using the control samples of each corresponding gestational age group (i.e. 12, 20, 28 and 36 weeks). Therefore they are not comparable across gestational age.

Longitudinal analysis

The method applied to the longitudinal analysis of cfRNA is described in the main paper and the corresponding R code is available from https://obsgynaecam.github.io/cell-free-rna-2024/.

In **Supplementary Figure 4-6**, samples of the same gestational age group were colored the same and those from the same participant were indicated in grey lines linked in each interval. The blue lines represent fitted (i.e. predicted) cfRNA abundance from the mixed effect models with two fixed terms (a linear and quadratic term of gestational age) and a random intercept. The grey area along the blue line represents 95% confidence interval of the fitted values. The symbol "r2m", next to the gene name, represents the marginal r^2 and they are from **Supplementary Data 11**. The 100 cfRNAs shown in **Supplementary Figure 4** and **5**, were selected from **Supplementary Data 11** column B (descending order of the marginal r^2) and H (ascending order of the combined p-value), respectively. The 100 cfRNAs shown in **Supplementary Figure 6** were selected from **Supplementary Data 12** column E (ascending order of the p-value) restricted to the negative values in the column D (coefficient of GA).

Supplementary Figure 4.

Top 100 cfRNAs

by the coefficient of determination

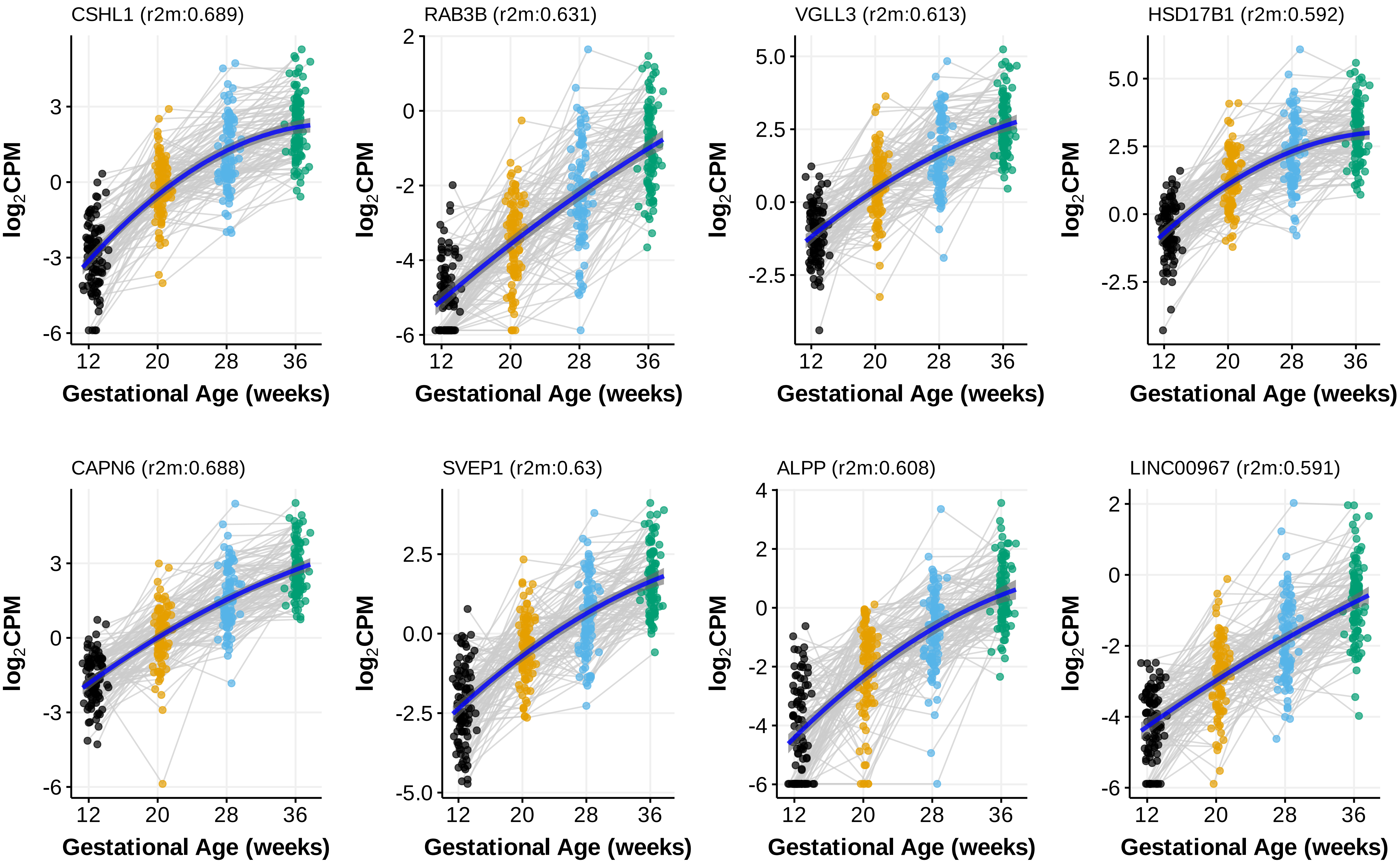
CSHL1 (r2m:0.689)
RAB3B (r2m:0.631)
VGLL3 (r2m:0.613)
HSD17B1 (r2m:0.592)
CAPN6 (r2m:0.688)
SVEP1 (r2m:0.63)
ALPP (r2m:0.608)
LINC00967 (r2m:0.591)
log_2CPM
Gestational Age (weeks)
12
20
28
36

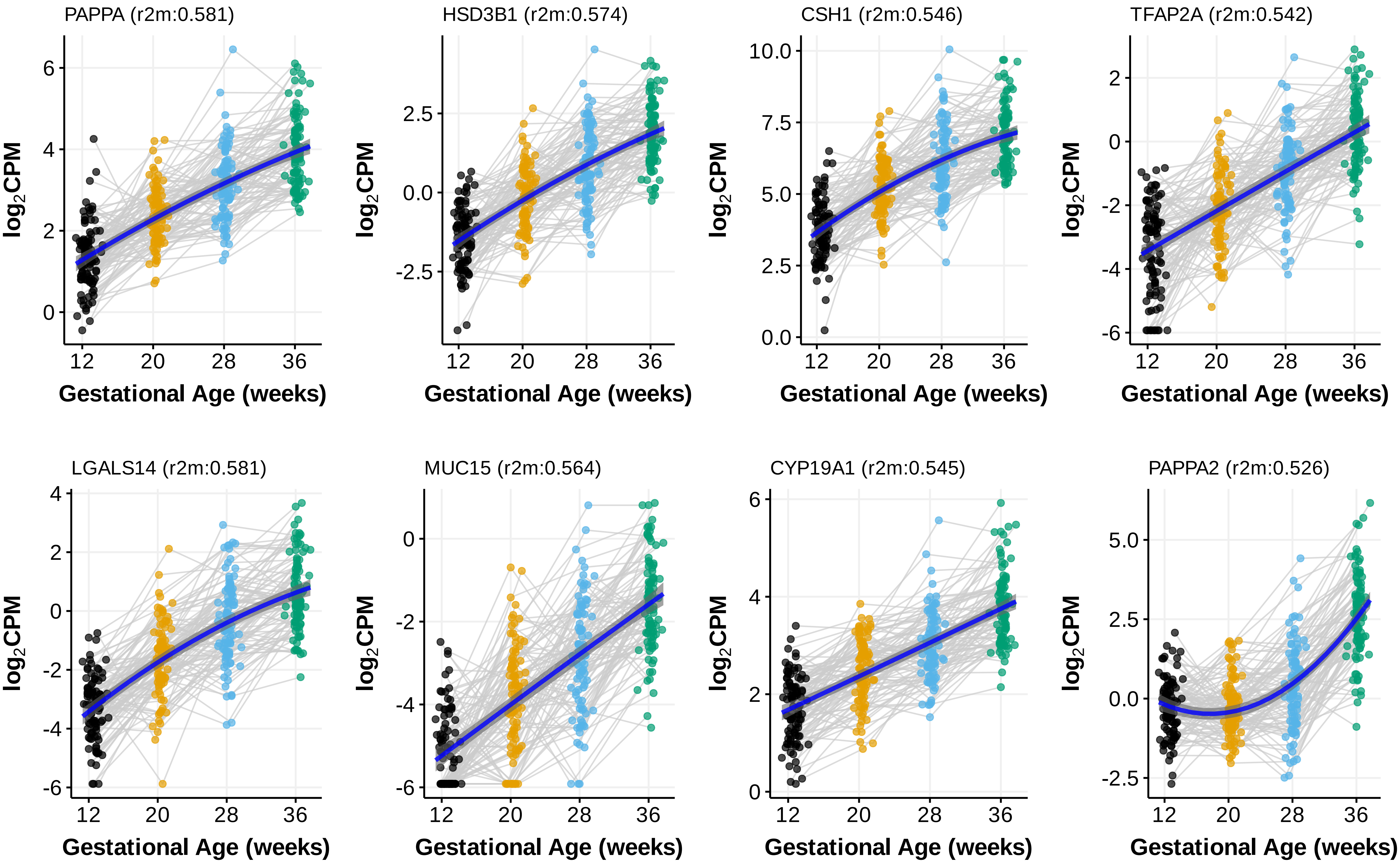
PAPPA (r2m:0.581)
HSD3B1 (r2m:0.574)
CSH1 (r2m:0.546)
TFAP2A (r2m:0.542)
LGALS14 (r2m:0.581)
MUC15 (r2m:0.564)
CYP19A1 (r2m:0.545)
PAPPA2 (r2m:0.526)
log2CPM
Gestational Age (weeks)
12
20
28
36

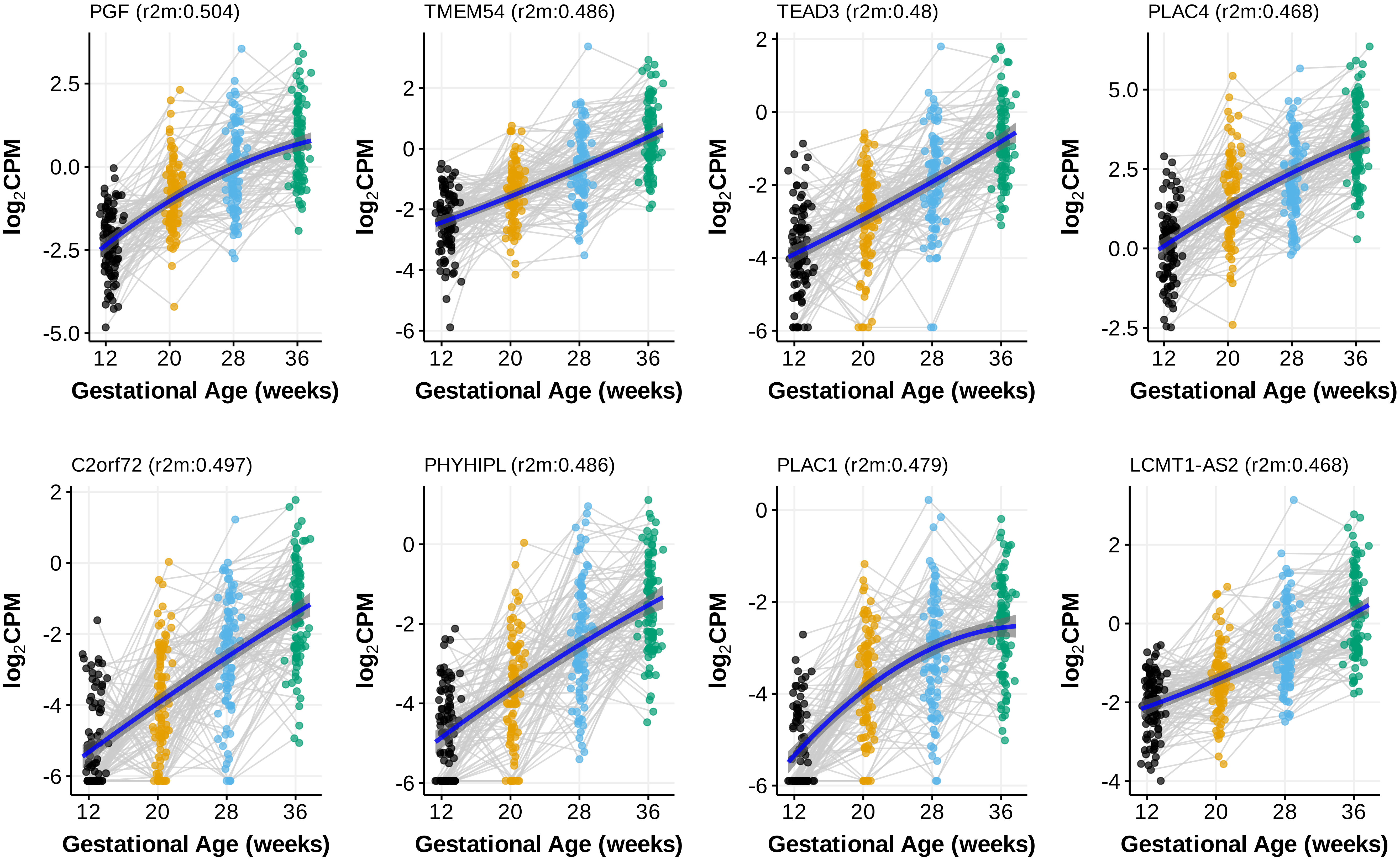
PGF (r2m:0.504)
TMEM54 (r2m:0.486)
TEAD3 (r2m:0.48)
PLAC4 (r2m:0.468)
C2orf72 (r2m:0.497)
PHYHIPL (r2m:0.486)
PLAC1 (r2m:0.479)
LCMT1-AS2 (r2m:0.468)
2.5
0.0
-2.5
-5.0
2
0
-2
-4
-6
5.0
12
20
28
36
log_2CPM
Gestational Age (weeks)

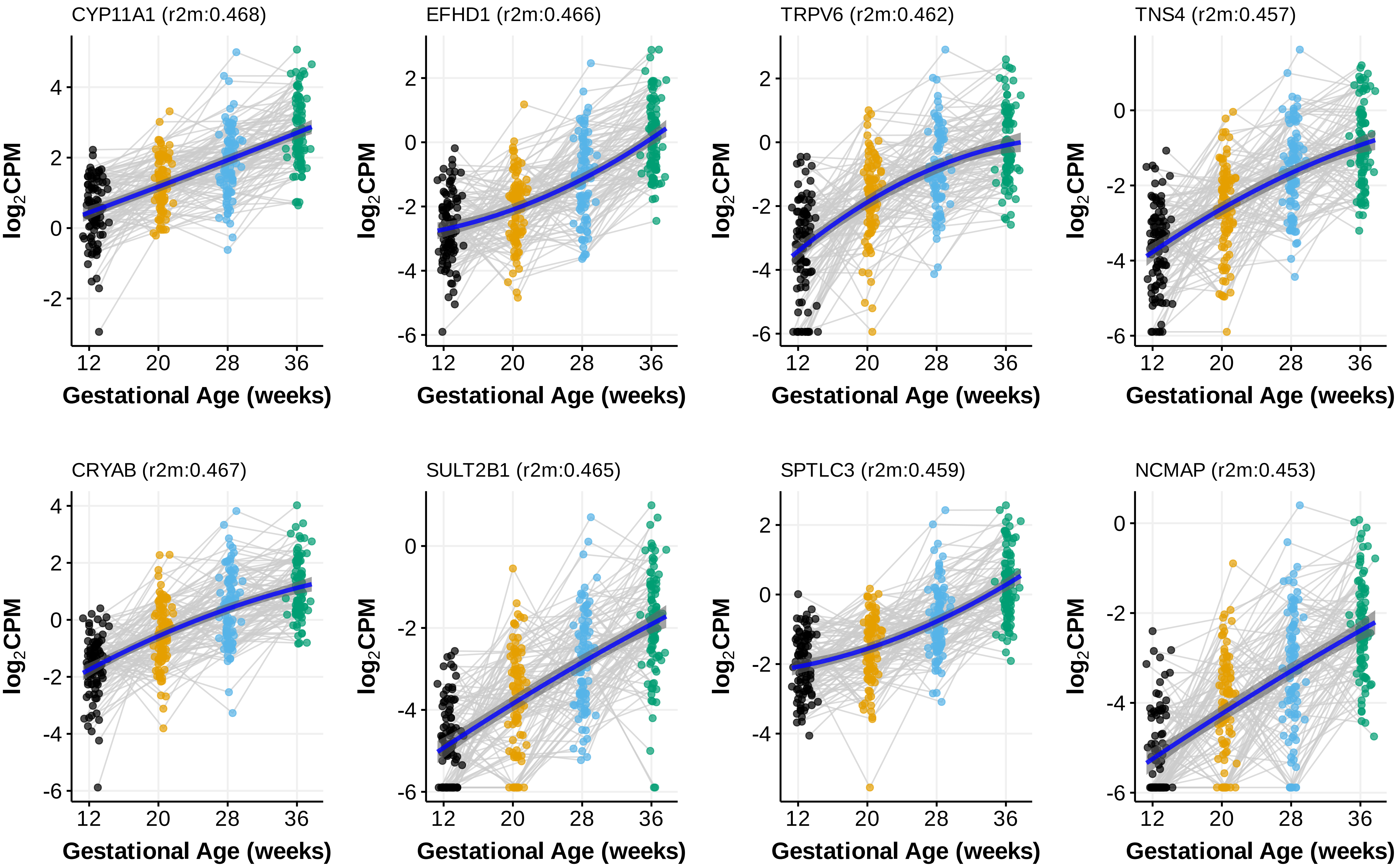
CYP11A1 (r2m:0.468)
EFHD1 (r2m:0.466)
TRPV6 (r2m:0.462)
TNS4 (r2m:0.457)
CRYAB (r2m:0.467)
SULT2B1 (r2m:0.465)
SPTLC3 (r2m:0.459)
NCMAP (r2m:0.453)
log_2CPM
Gestational Age (weeks)
12
20
28
36

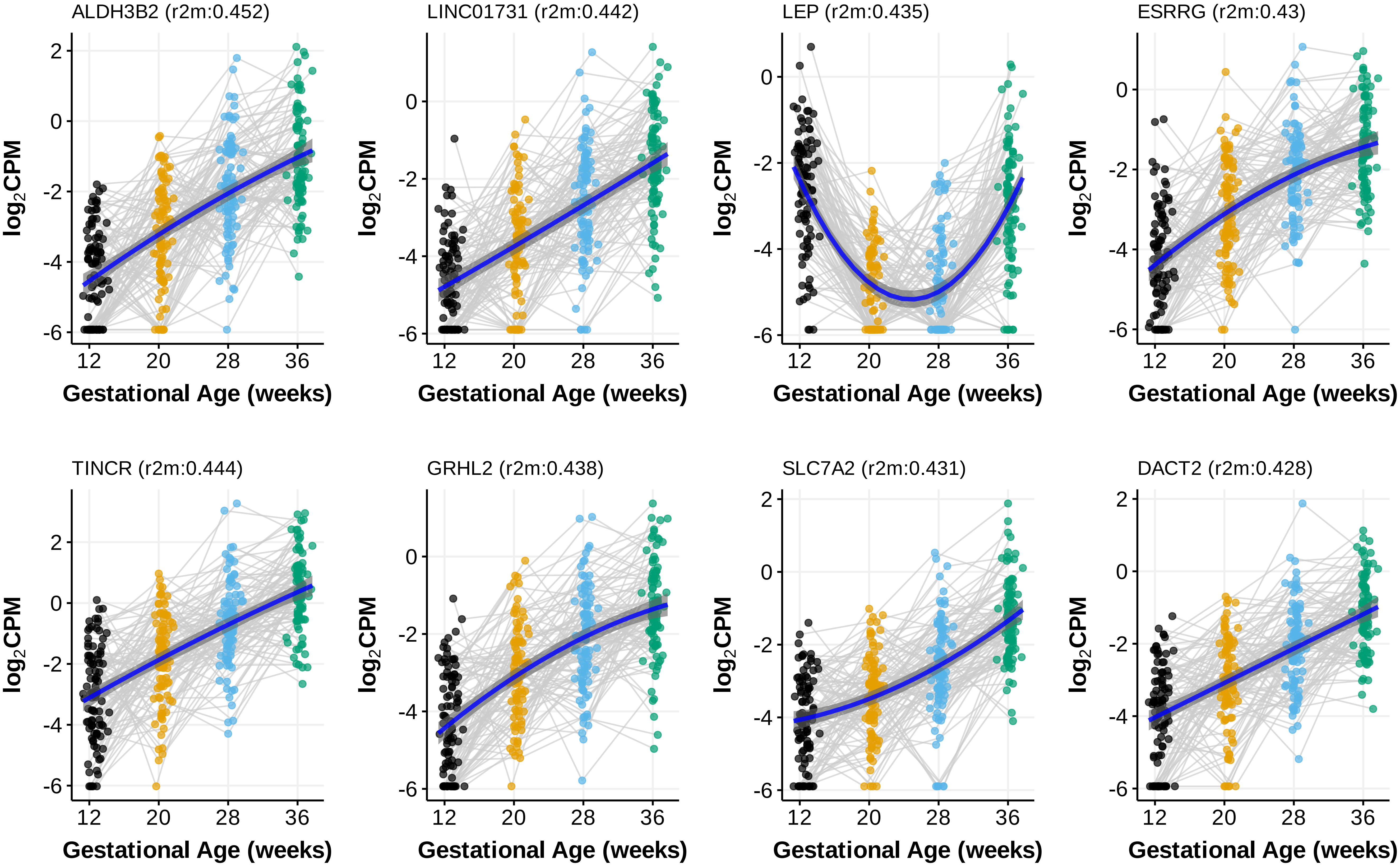

ALDH3B2 (r2m:0.452)
LINC01731 (r2m:0.442)
LEP (r2m:0.435)
ESRRG (r2m:0.43)
TINCR (r2m:0.444)
GRHL2 (r2m:0.438)
SLC7A2 (r2m:0.431)
DACT2 (r2m:0.428)
log_2CPM
Gestational Age (weeks)
12
20
28
36

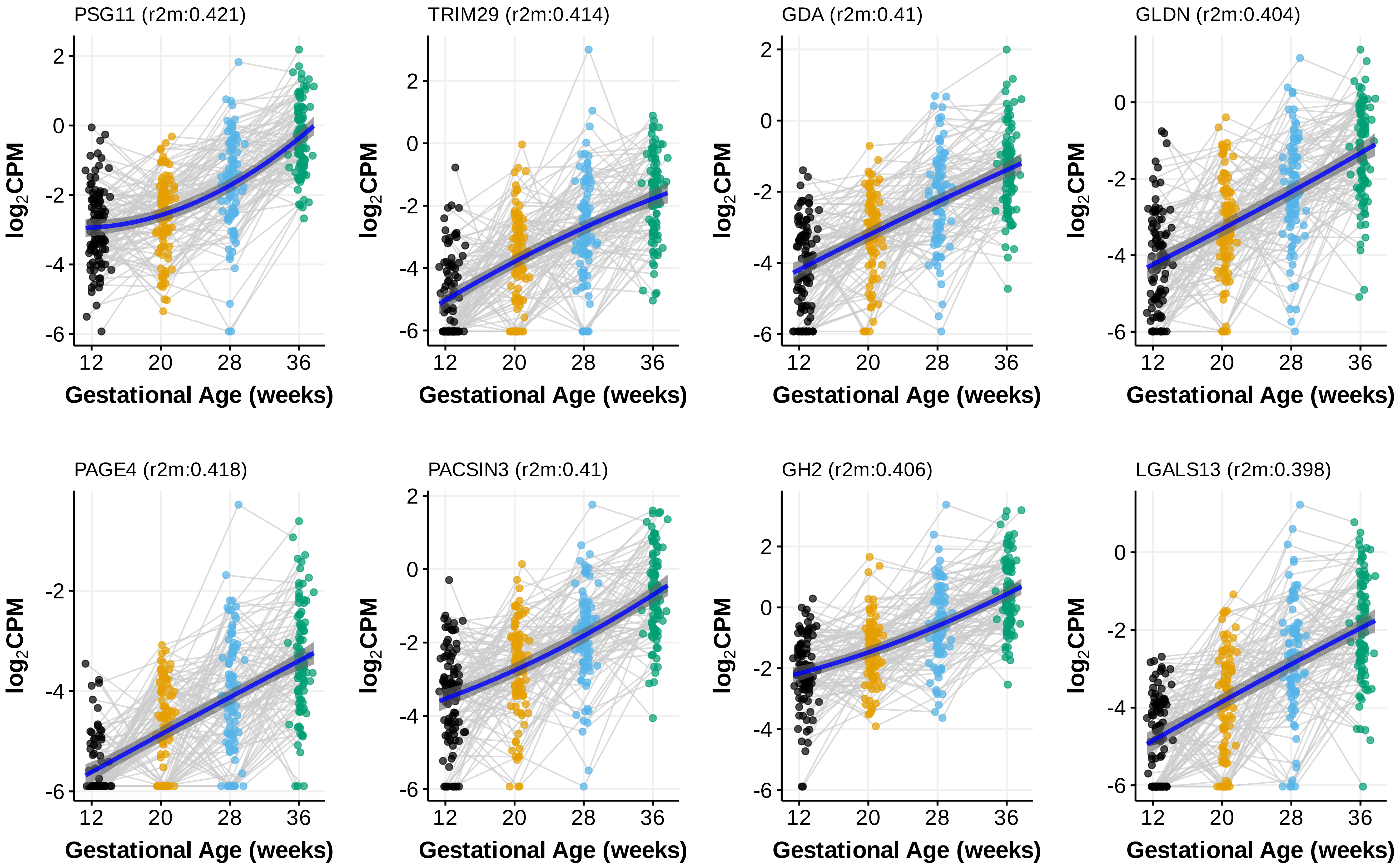

PSG11 (r2m:0.421)
TRIM29 (r2m:0.414)
GDA (r2m:0.41)
GLDN (r2m:0.404)
PAGE4 (r2m:0.418)
PACSIN3 (r2m:0.41)
GH2 (r2m:0.406)
LGALS13 (r2m:0.398)
log_2CPM
Gestational Age (weeks)
12
20
28
36

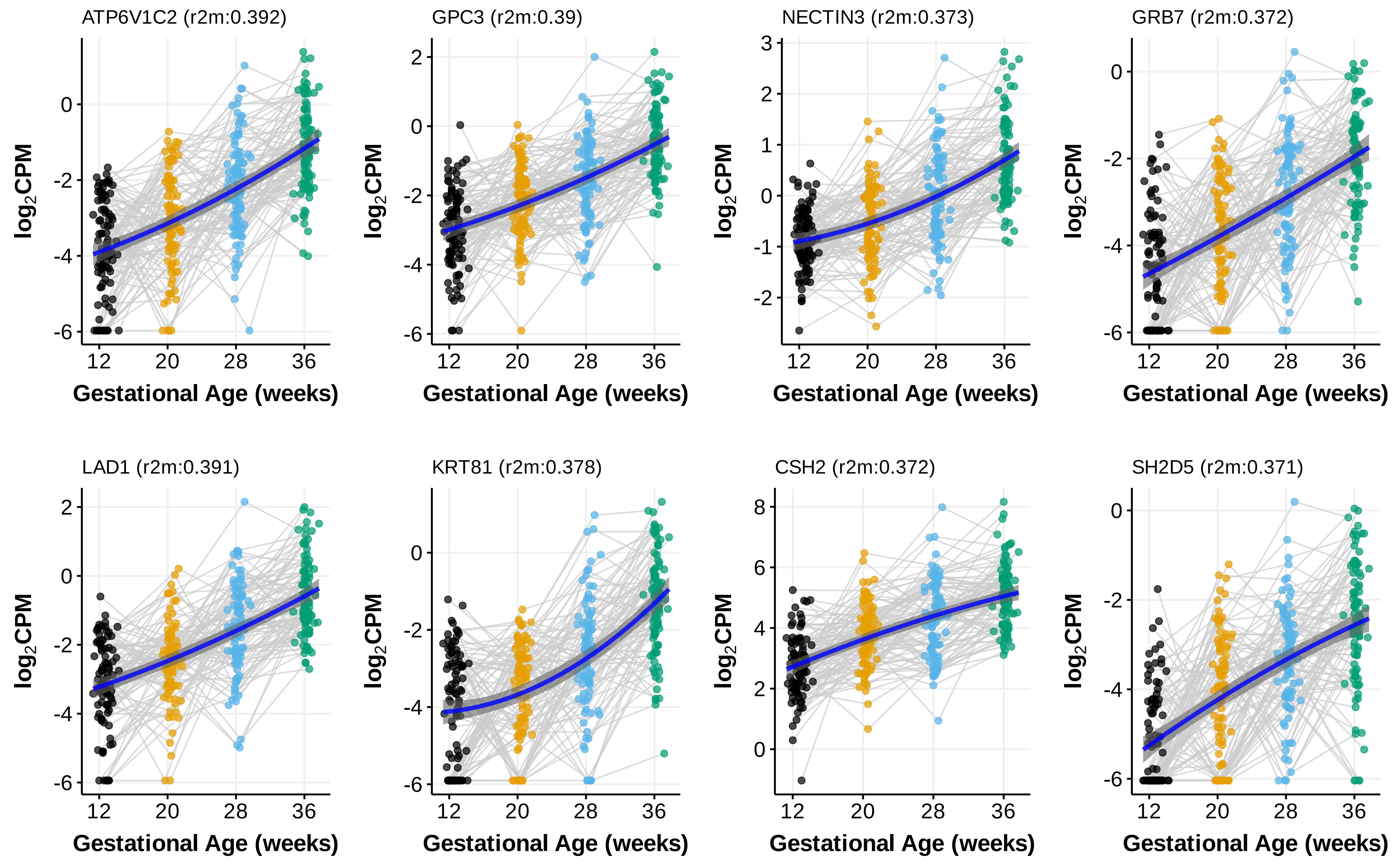

ATP6V1C2 (r2m:0.392)
GPC3 (r2m:0.39)
NECTIN3 (r2m:0.373)
GRB7 (r2m:0.372)
LAD1 (r2m:0.391)
KRT81 (r2m:0.378)
CSH2 (r2m:0.372)
SH2D5 (r2m:0.371)
log2CPM
Gestational Age (weeks)
12
20
28
36

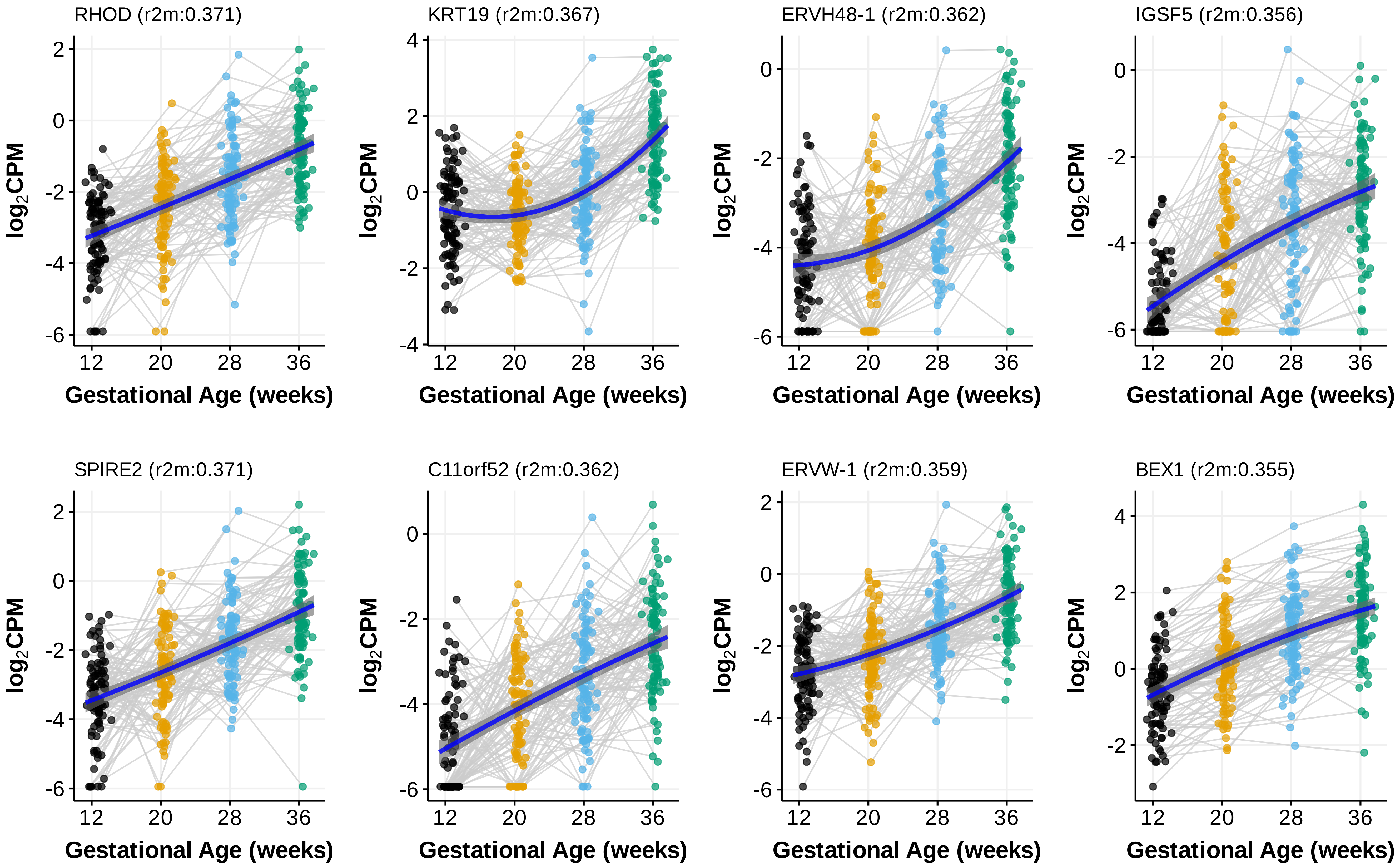
RHOD (r2m:0.371)
KRT19 (r2m:0.367)
ERVH48-1 (r2m:0.362)
IGSF5 (r2m:0.356)
SPIRE2 (r2m:0.371)
C11orf52 (r2m:0.362)
ERVW-1 (r2m:0.359)
BEX1 (r2m:0.355)
log2CPM
Gestational Age (weeks)
12
20
28
36

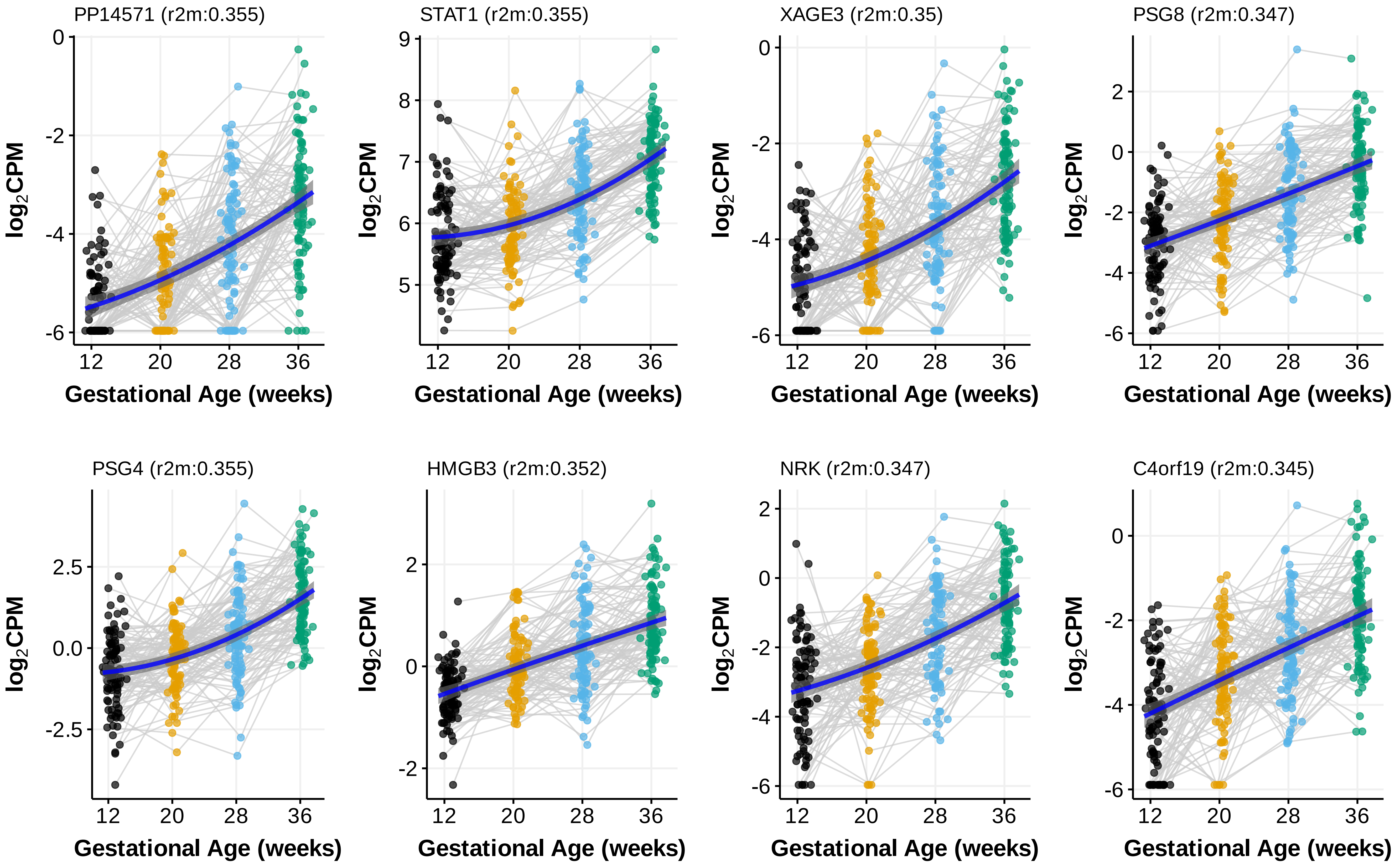

PP14571 (r2m:0.355)
STAT1 (r2m:0.355)
XAGE3 (r2m:0.35)
PSG8 (r2m:0.347)
PSG4 (r2m:0.355)
HMGB3 (r2m:0.352)
NRK (r2m:0.347)
C4orf19 (r2m:0.345)
log_2CPM
Gestational Age (weeks)
12
20
28
36

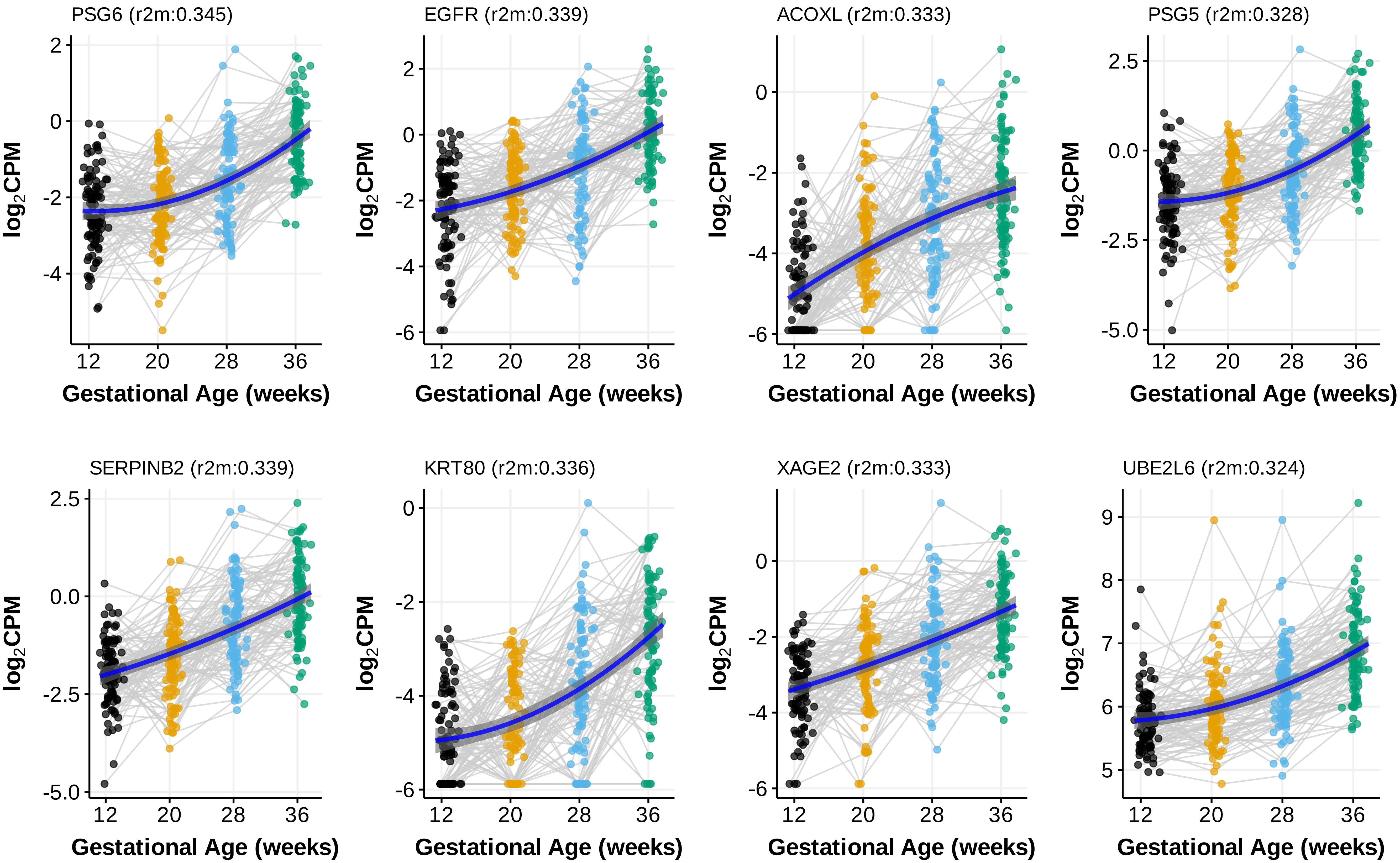

PSG6 (r2m:0.345)
EGFR (r2m:0.339)
ACOXL (r2m:0.333)
PSG5 (r2m:0.328)
SERPINB2 (r2m:0.339)
KRT80 (r2m:0.336)
XAGE2 (r2m:0.333)
UBE2L6 (r2m:0.324)
log2CPM
Gestational Age (weeks)
12
20
28
36

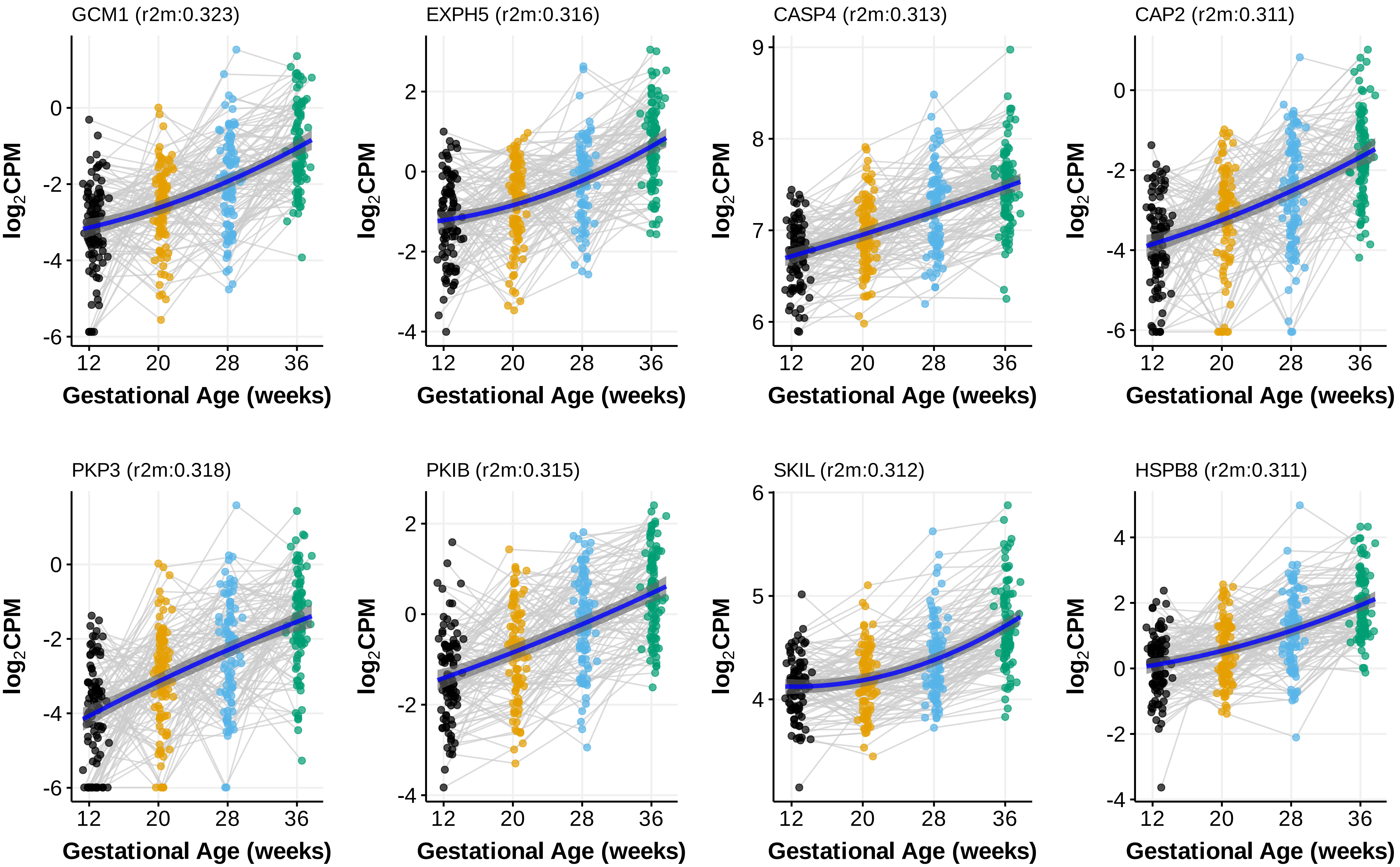
GCM1 (r2m:0.323)
EXPH5 (r2m:0.316)
CASP4 (r2m:0.313)
CAP2 (r2m:0.311)
PKP3 (r2m:0.318)
PKIB (r2m:0.315)
SKIL (r2m:0.312)
HSPB8 (r2m:0.311)
log2CPM
Gestational Age (weeks)
12
20
28
36

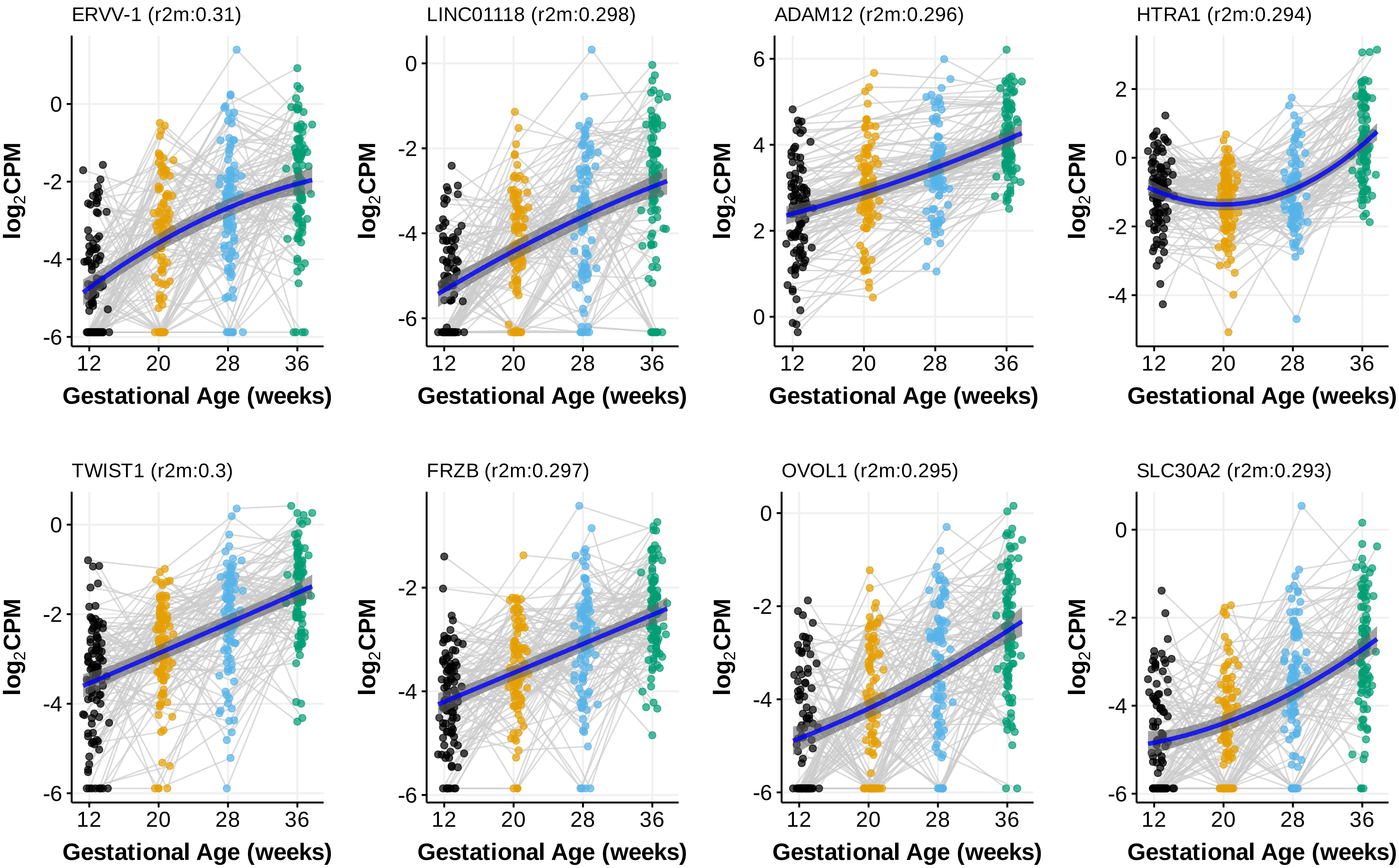
ERVV-1 (r2m:0.31)
LINC01118 (r2m:0.298)
ADAM12 (r2m:0.296)
HTRA1 (r2m:0.294)
TWIST1 (r2m:0.3)
FRZB (r2m:0.297)
OVOL1 (r2m:0.295)
SLC30A2 (r2m:0.293)
log2CPM
Gestational Age (weeks)
12
20
28
36

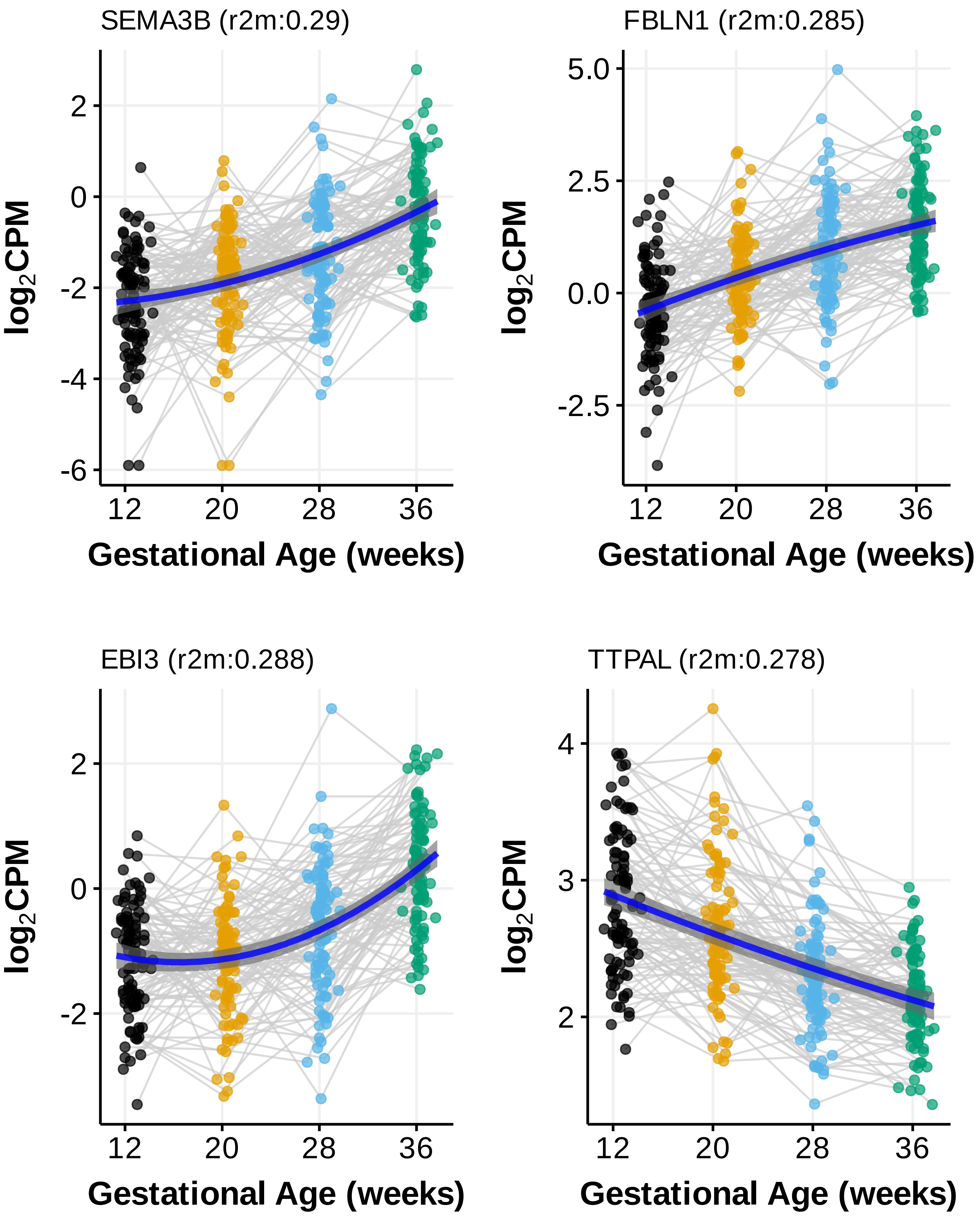

SEMA3B (r2m:0.29)
FBLN1 (r2m:0.285)
EBI3 (r2m:0.288)
TTPAL (r2m:0.278)
$\log_2$CPM
Gestational Age (weeks)
12
20
28
36
2
0
-2
-4
-6
5.0
2.5
0.0
-2.5
4
3

Supplementary Figure 5.
Top 100 cfRNAs
by the combined p-values

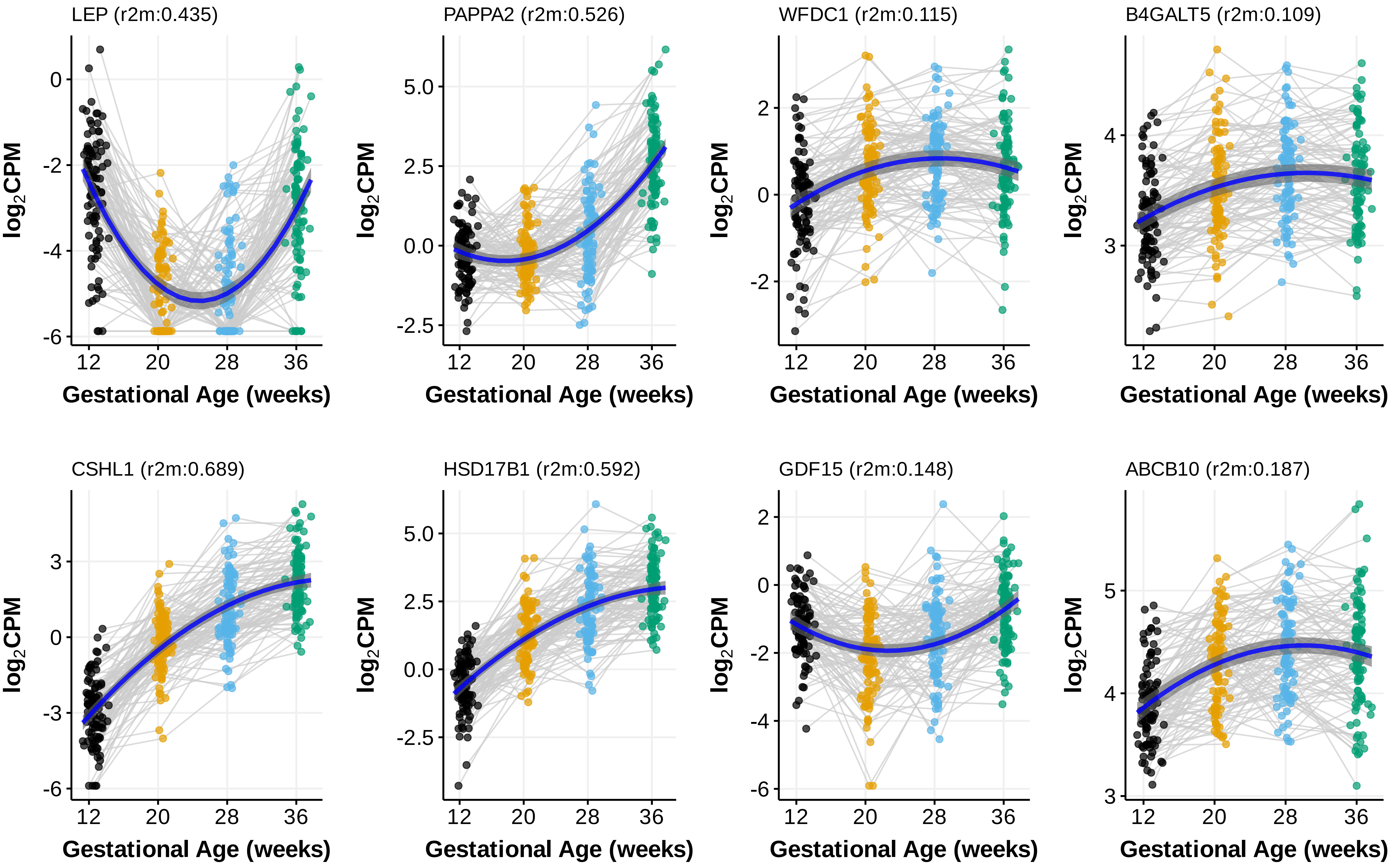

LEP (r2m:0.435)
PAPPA2 (r2m:0.526)
WFDC1 (r2m:0.115)
B4GALT5 (r2m:0.109)
CSHL1 (r2m:0.689)
HSD17B1 (r2m:0.592)
GDF15 (r2m:0.148)
ABCB10 (r2m:0.187)
$\log_2$CPM
Gestational Age (weeks)
12
20
28
36

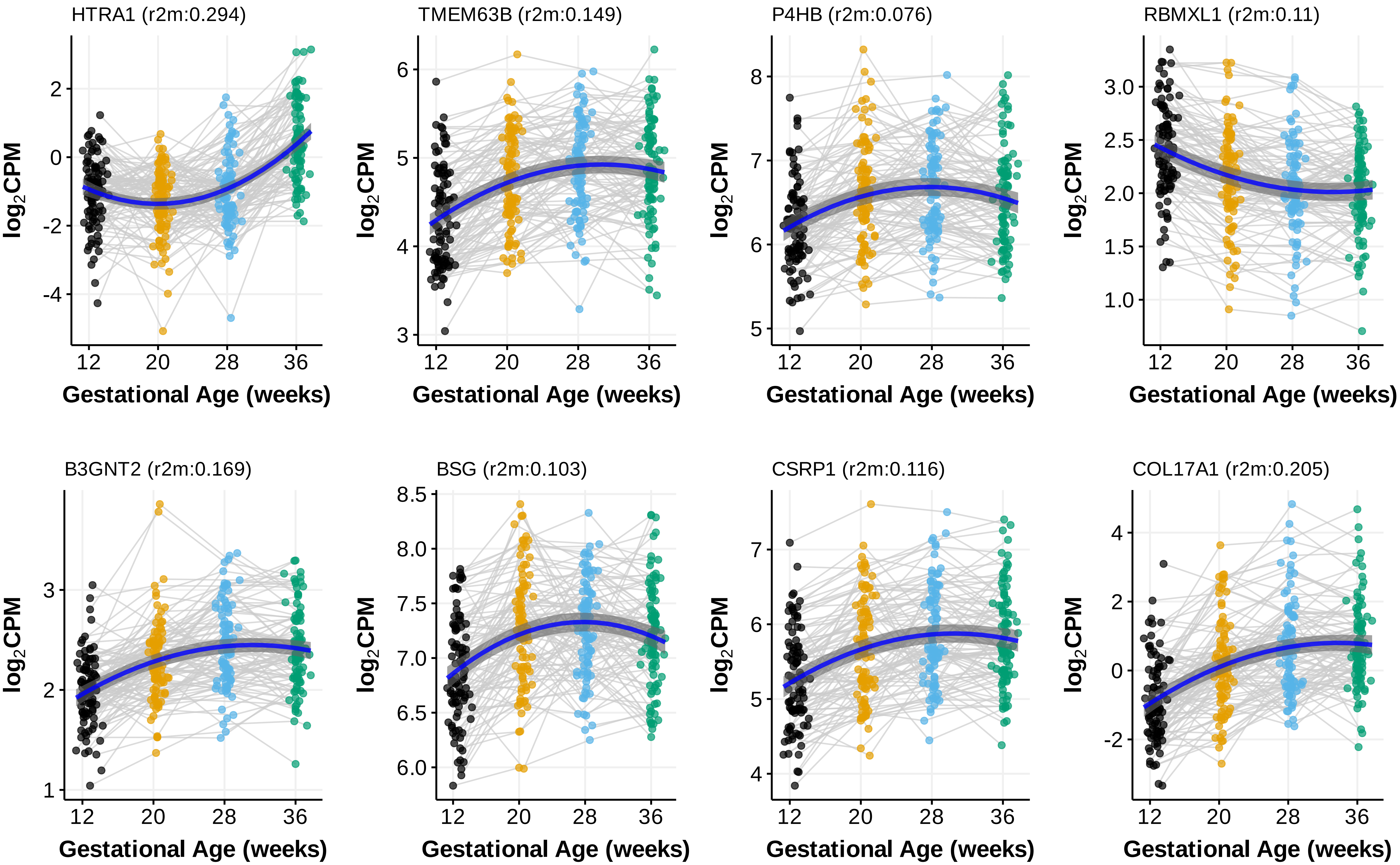

HTRA1 (r2m:0.294)
TMEM63B (r2m:0.149)
P4HB (r2m:0.076)
RBMXL1 (r2m:0.11)
B3GNT2 (r2m:0.169)
BSG (r2m:0.103)
CSRP1 (r2m:0.116)
COL17A1 (r2m:0.205)
$\log_2$CPM
Gestational Age (weeks)
12
20
28
36

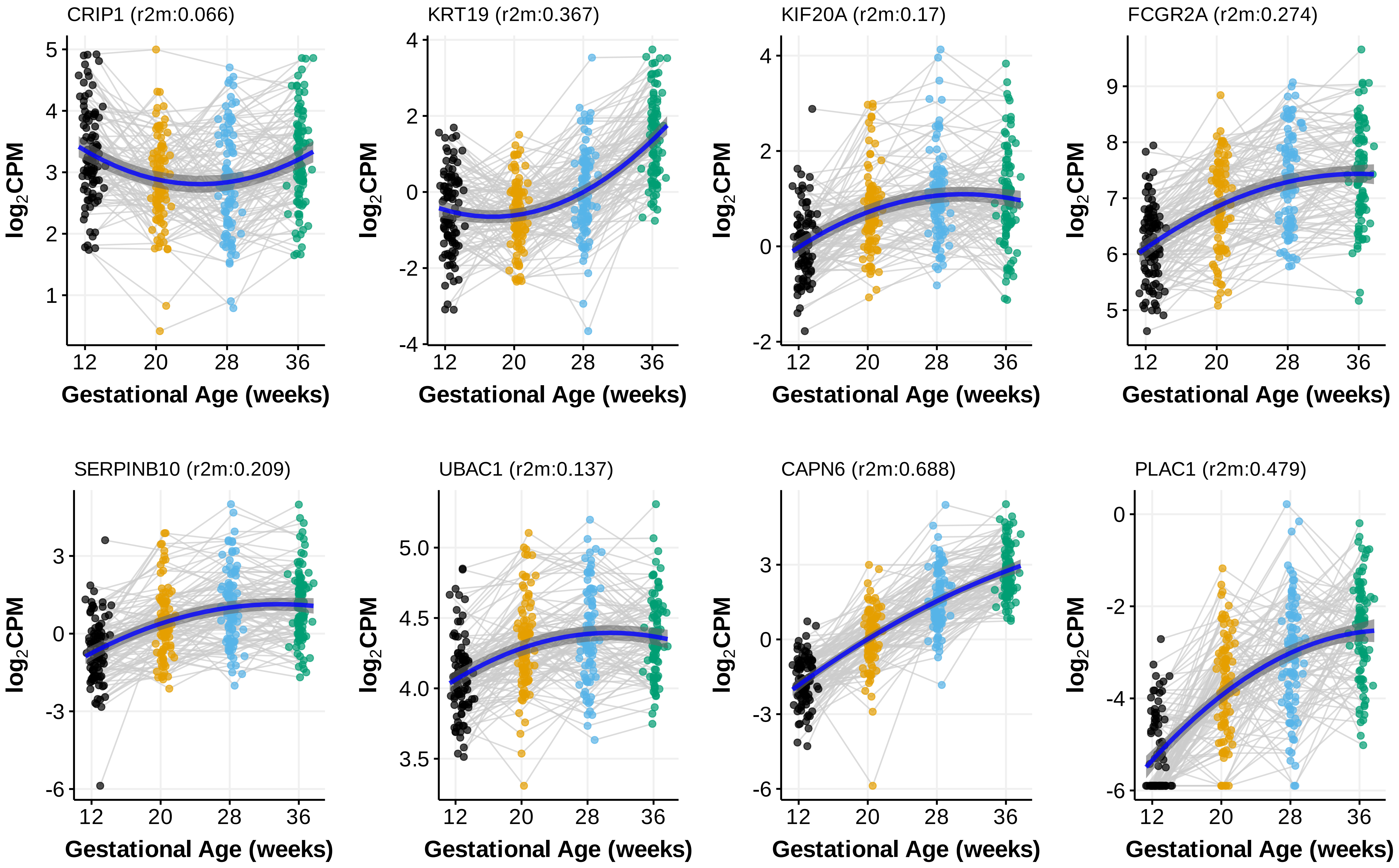

CRIP1 (r2m:0.066)
KRT19 (r2m:0.367)
KIF20A (r2m:0.17)
FCGR2A (r2m:0.274)
SERPINB10 (r2m:0.209)
UBAC1 (r2m:0.137)
CAPN6 (r2m:0.688)
PLAC1 (r2m:0.479)
log2CPM
Gestational Age (weeks)
12
20
28
36

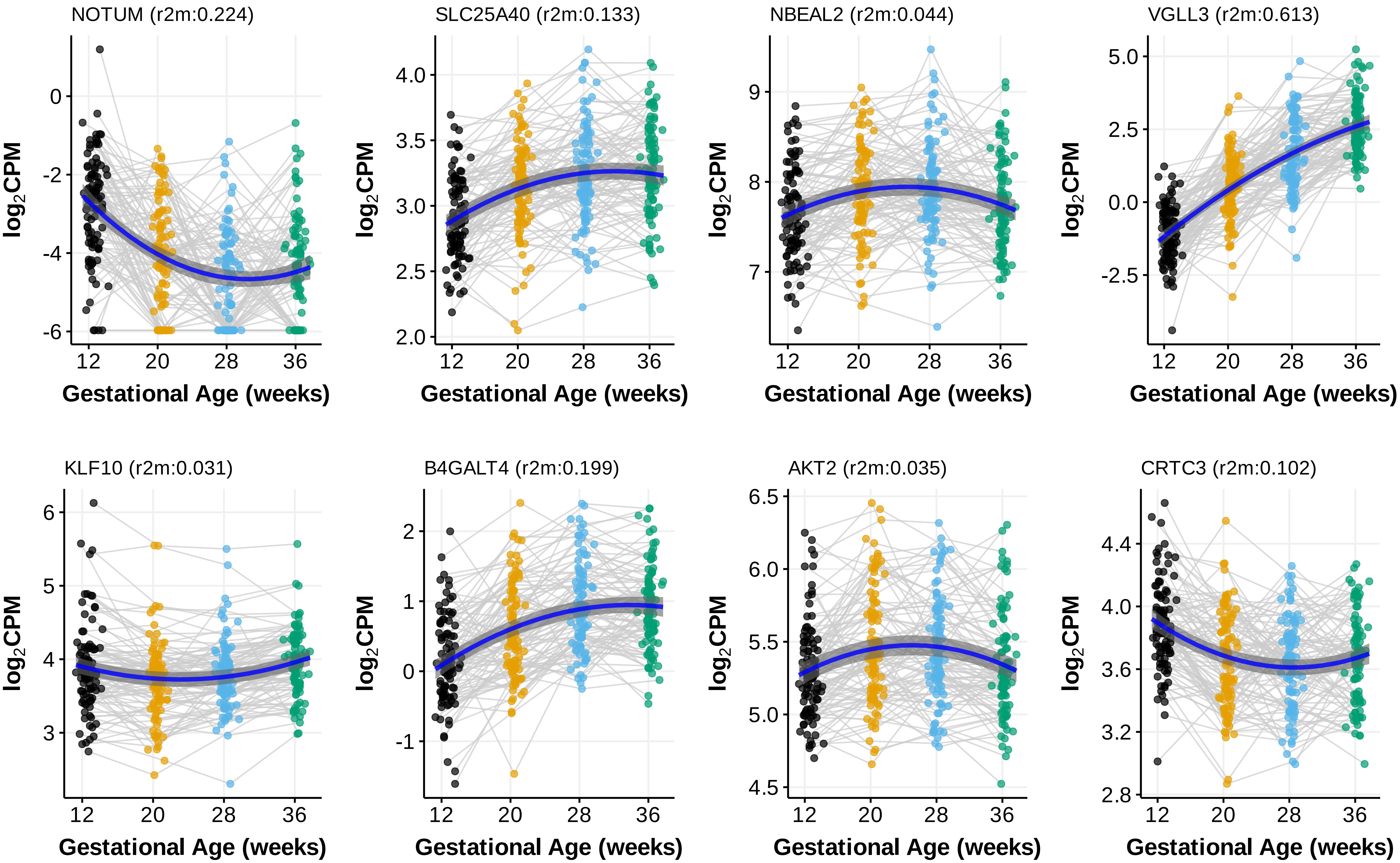

NOTUM (r2m:0.224)
SLC25A40 (r2m:0.133)
NBEAL2 (r2m:0.044)
VGLL3 (r2m:0.613)
KLF10 (r2m:0.031)
B4GALT4 (r2m:0.199)
AKT2 (r2m:0.035)
CRTC3 (r2m:0.102)
log2CPM
Gestational Age (weeks)
12
20
28
36

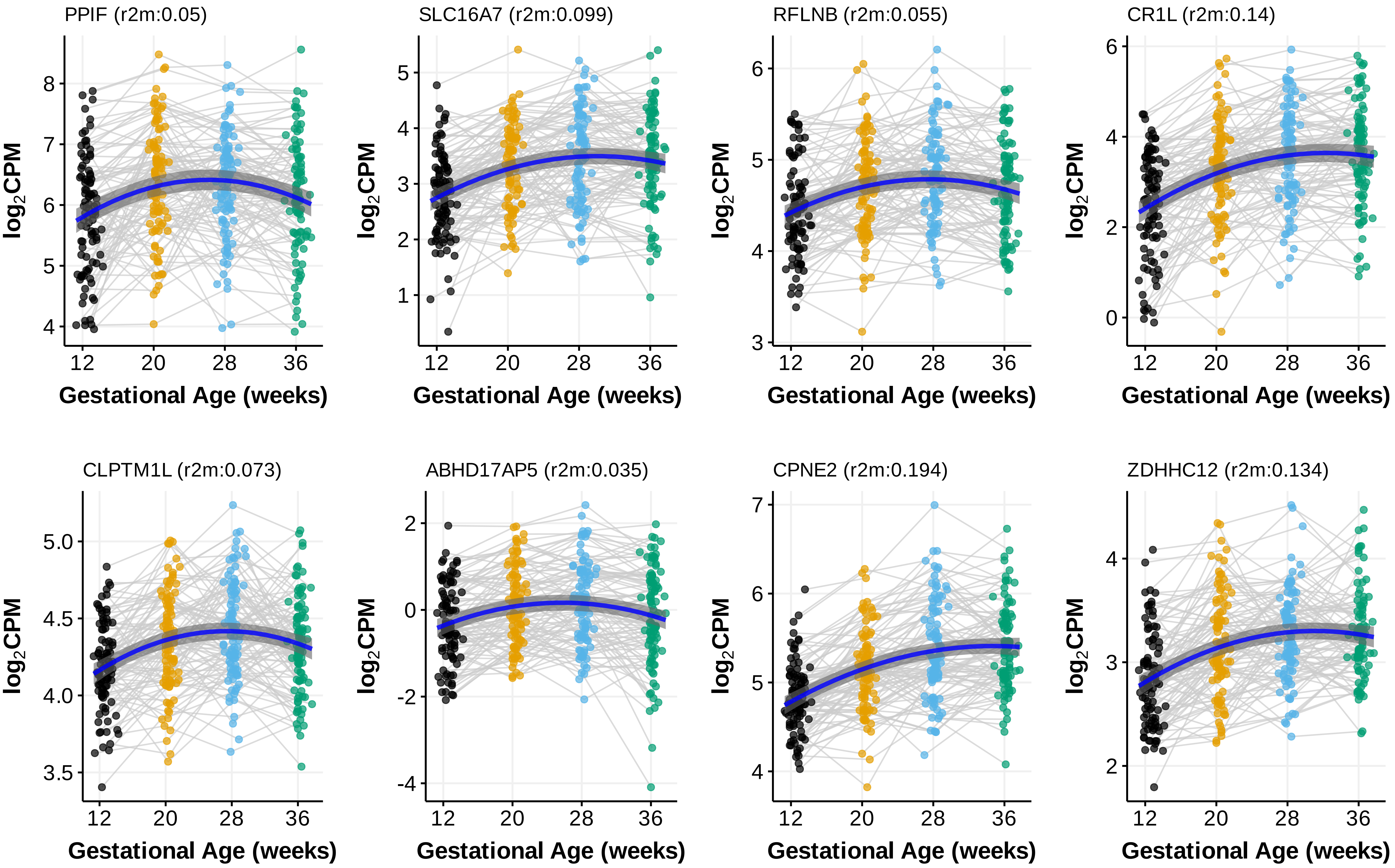
PPIF (r2m:0.05)
SLC16A7 (r2m:0.099)
RFLNB (r2m:0.055)
CR1L (r2m:0.14)
CLPTM1L (r2m:0.073)
ABHD17AP5 (r2m:0.035)
CPNE2 (r2m:0.194)
ZDHHC12 (r2m:0.134)
log2CPM
Gestational Age (weeks)
12
20
28
36

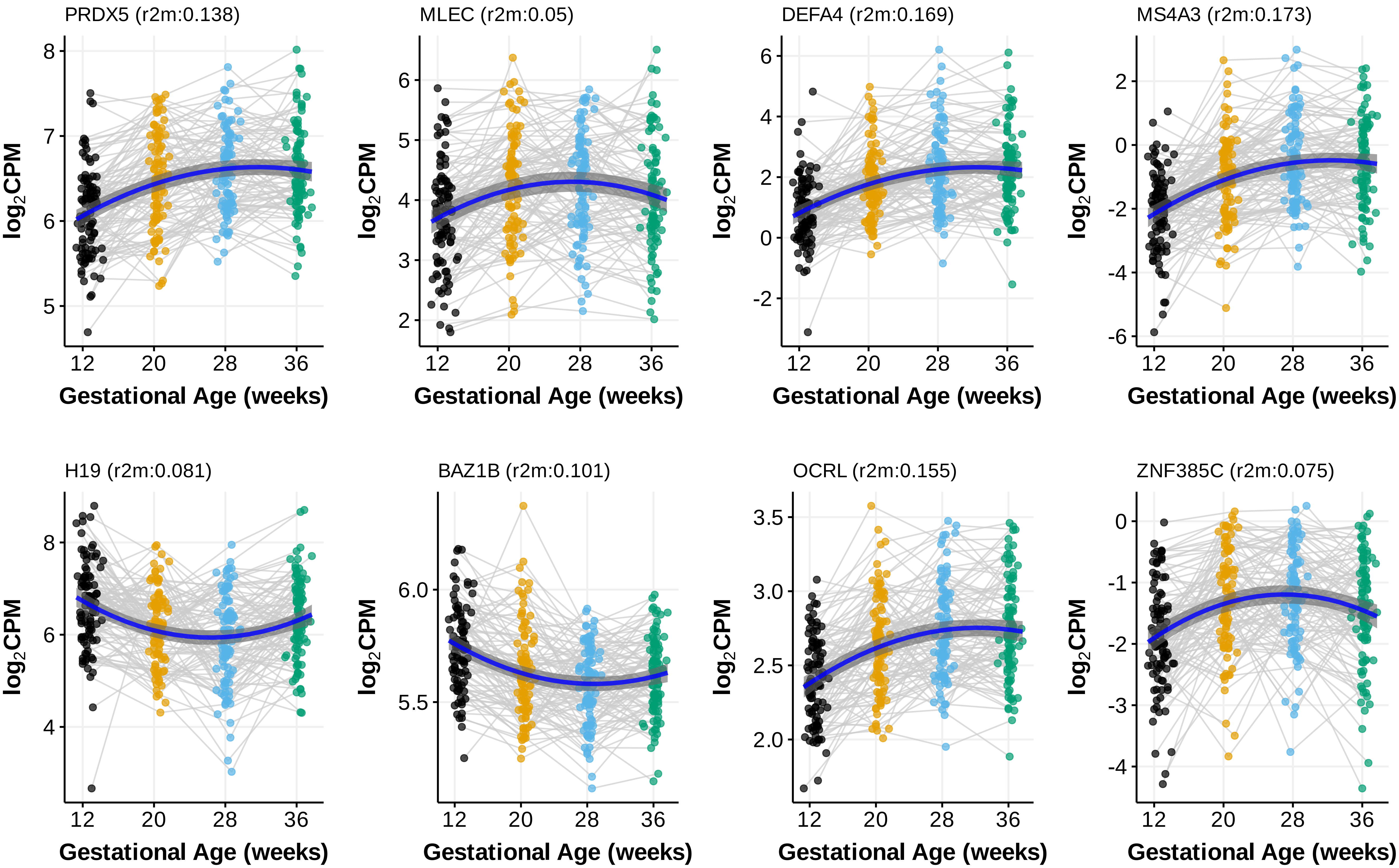

PRDX5 (r2m:0.138)
MLEC (r2m:0.05)
DEFA4 (r2m:0.169)
MS4A3 (r2m:0.173)
H19 (r2m:0.081)
BAZ1B (r2m:0.101)
OCRL (r2m:0.155)
ZNF385C (r2m:0.075)
log_2CPM
Gestational Age (weeks)
12
20
28
36

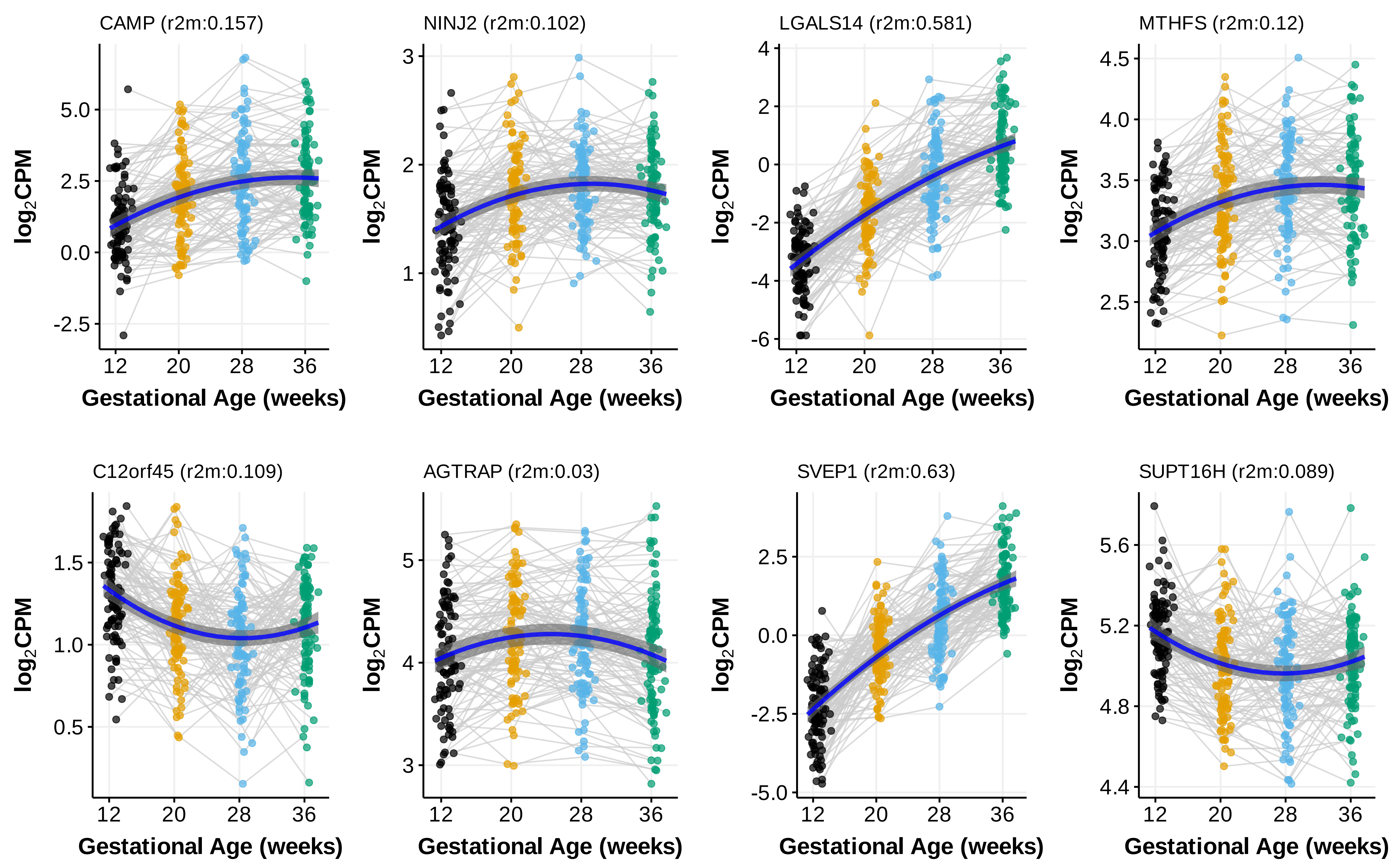
CAMP (r2m:0.157)
NINJ2 (r2m:0.102)
LGALS14 (r2m:0.581)
MTHFS (r2m:0.12)
C12orf45 (r2m:0.109)
AGTRAP (r2m:0.03)
SVEP1 (r2m:0.63)
SUPT16H (r2m:0.089)
log2CPM
Gestational Age (weeks)
12
20
28
36

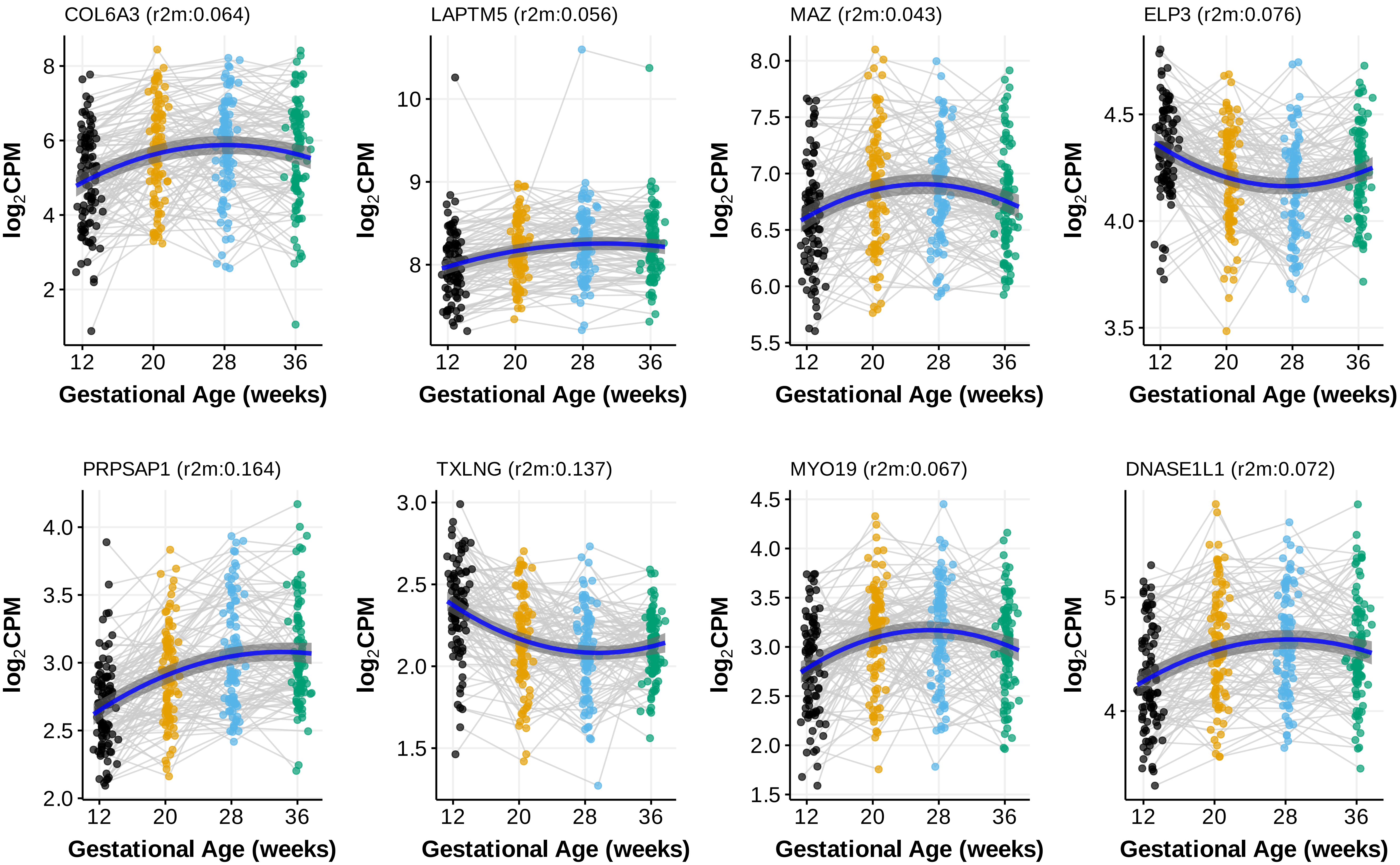

COL6A3 (r2m:0.064)
LAPTM5 (r2m:0.056)
MAZ (r2m:0.043)
ELP3 (r2m:0.076)
PRPSAP1 (r2m:0.164)
TXLNG (r2m:0.137)
MYO19 (r2m:0.067)
DNASE1L1 (r2m:0.072)
log2CPM
Gestational Age (weeks)
12
20
28
36

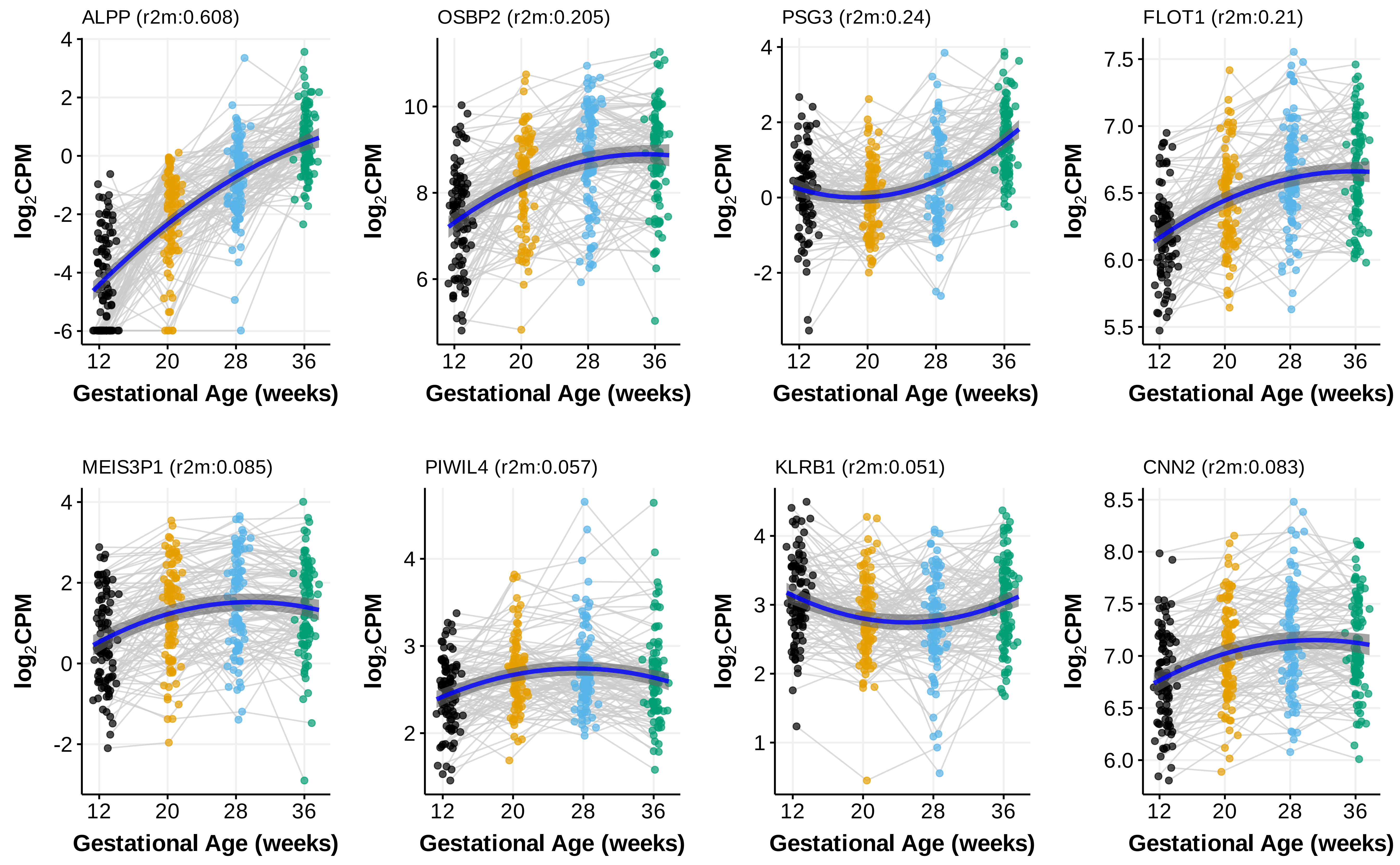
ALPP (r2m:0.608)
OSBP2 (r2m:0.205)
PSG3 (r2m:0.24)
FLOT1 (r2m:0.21)
MEIS3P1 (r2m:0.085)
PIWIL4 (r2m:0.057)
KLRB1 (r2m:0.051)
CNN2 (r2m:0.083)
log2CPM
Gestational Age (weeks)
12
20
28
36

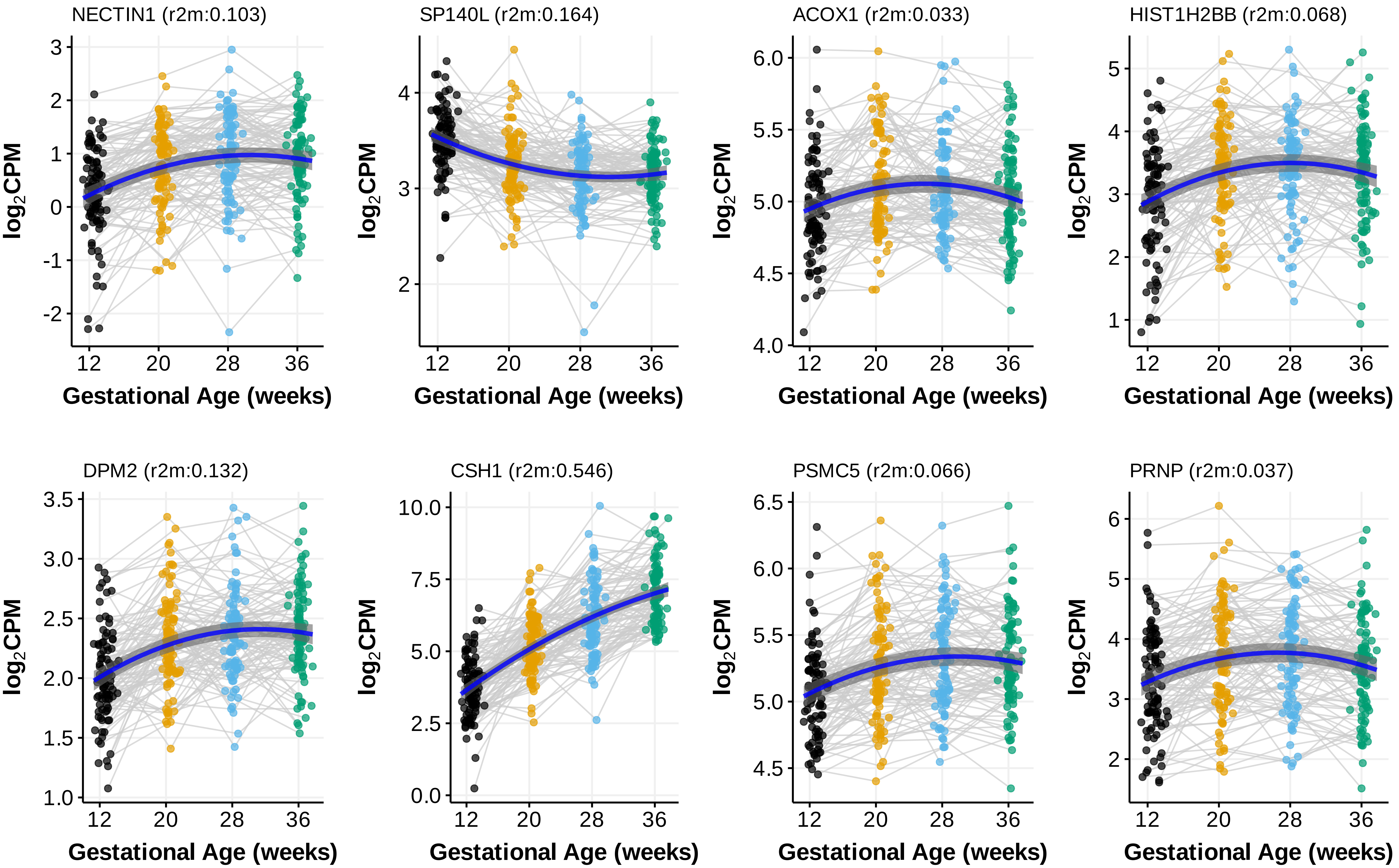

NECTIN1 (r2m:0.103)
SP140L (r2m:0.164)
ACOX1 (r2m:0.033)
HIST1H2BB (r2m:0.068)
DPM2 (r2m:0.132)
CSH1 (r2m:0.546)
PSMC5 (r2m:0.066)
PRNP (r2m:0.037)
log_2CPM
Gestational Age (weeks)
12
20
28
36

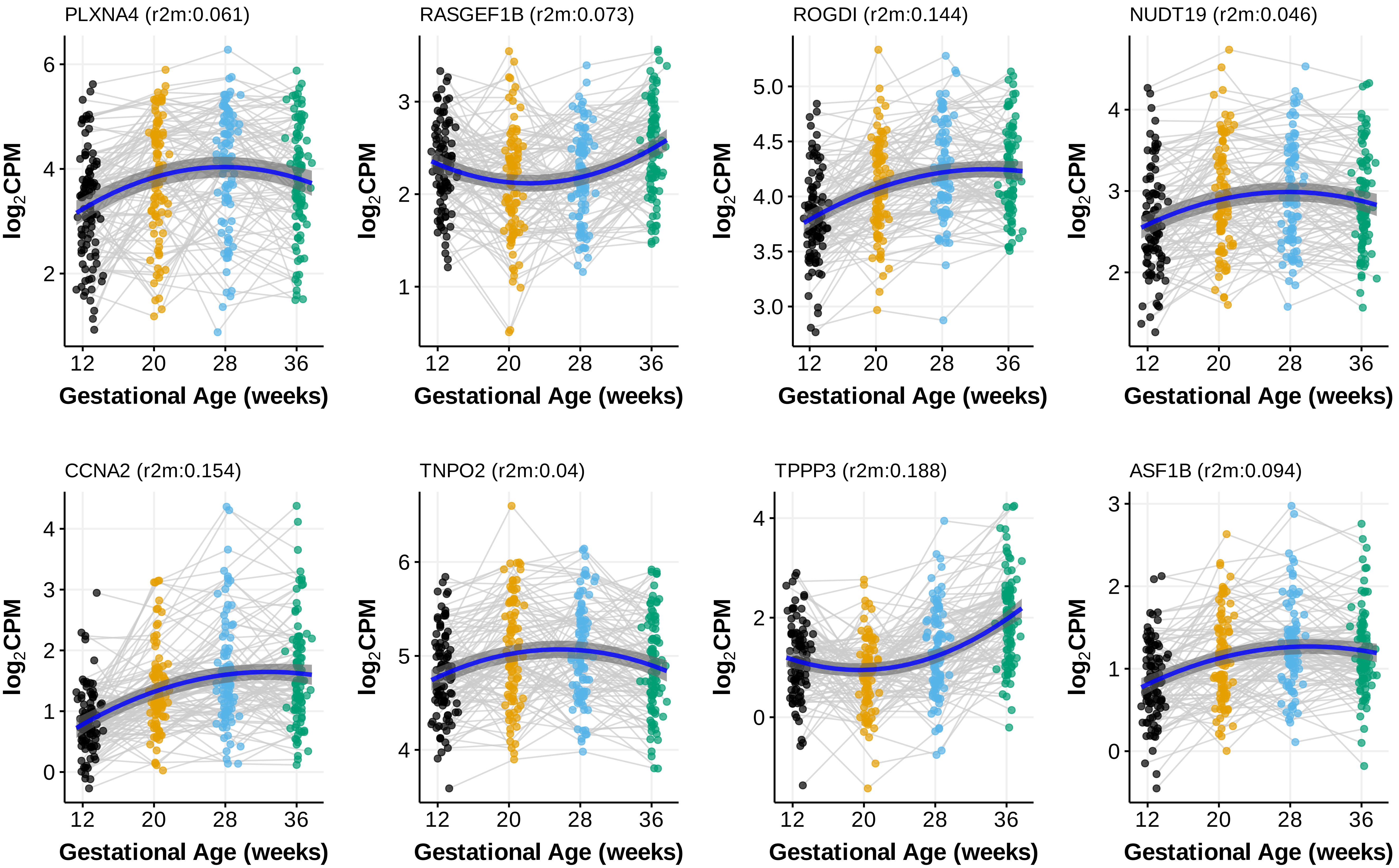
PLXNA4 (r2m:0.061)
RASGEF1B (r2m:0.073)
ROGDI (r2m:0.144)
NUDT19 (r2m:0.046)
CCNA2 (r2m:0.154)
TNPO2 (r2m:0.04)
TPPP3 (r2m:0.188)
ASF1B (r2m:0.094)
log2CPM
Gestational Age (weeks)
12
20
28
36

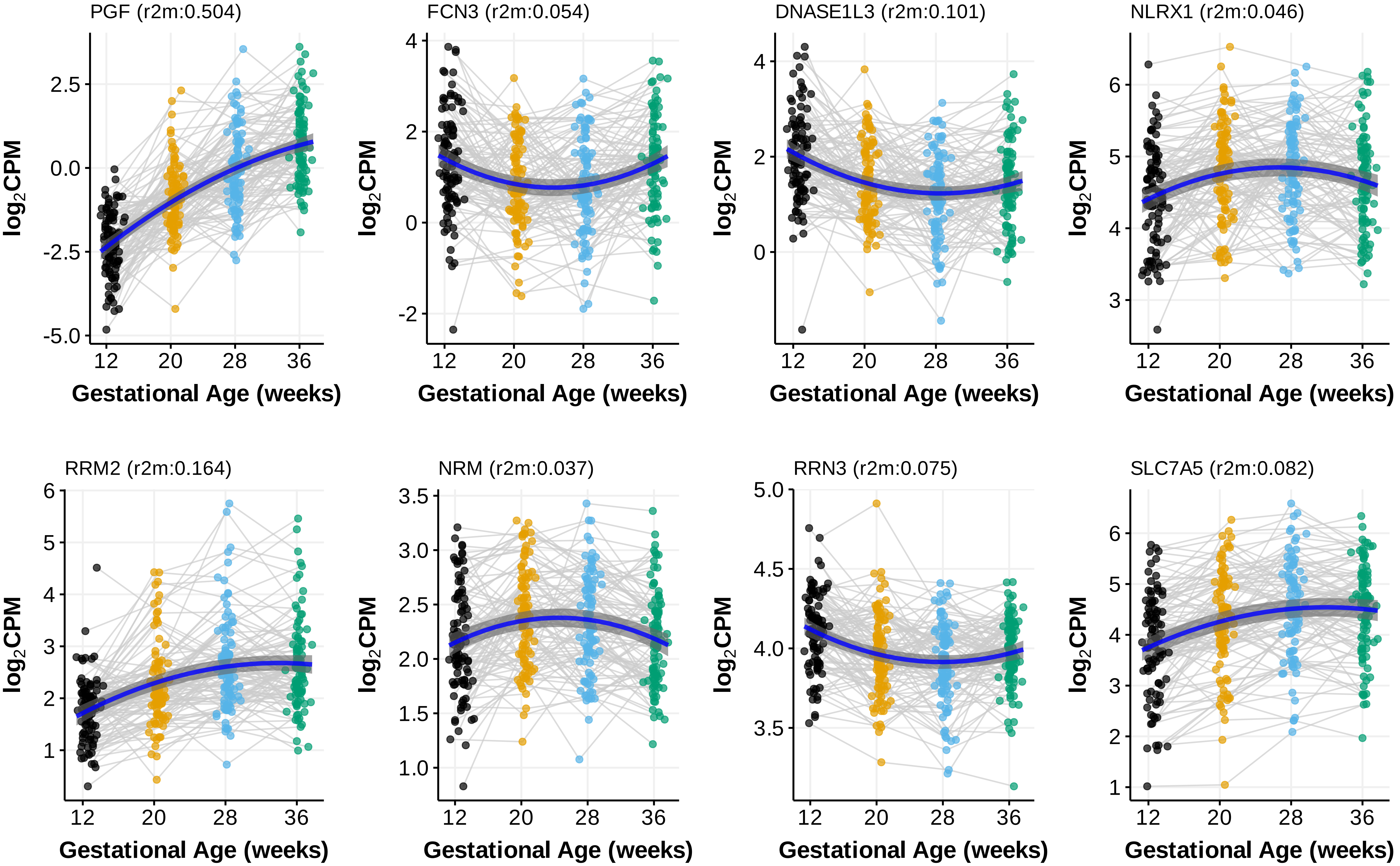
PGF (r2m:0.504)
FCN3 (r2m:0.054)
DNASE1L3 (r2m:0.101)
NLRX1 (r2m:0.046)
RRM2 (r2m:0.164)
NRM (r2m:0.037)
RRN3 (r2m:0.075)
SLC7A5 (r2m:0.082)
log2CPM
Gestational Age (weeks)
12
20
28
36

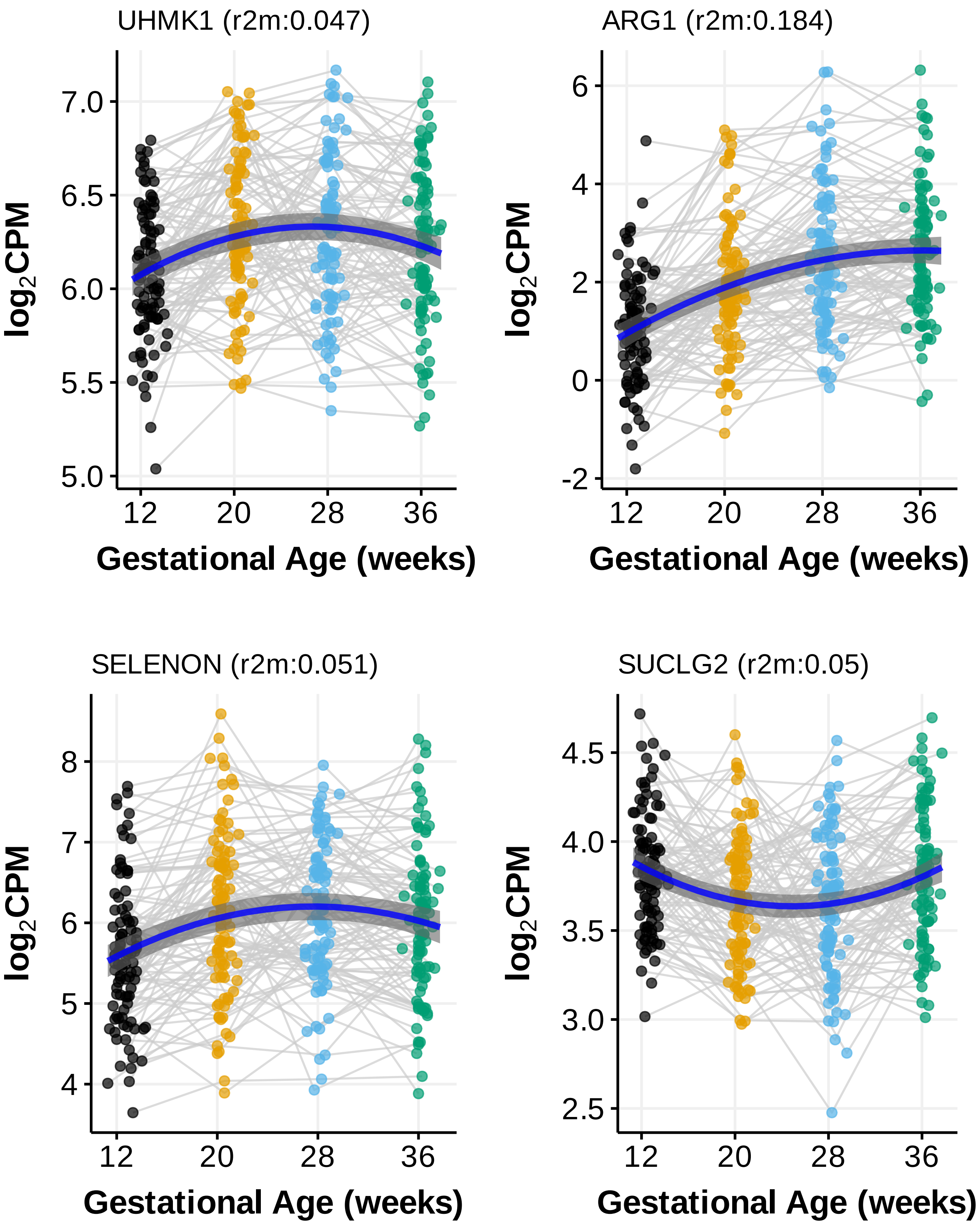

UHMK1 (r2m:0.047)
ARG1 (r2m:0.184)
SELENON (r2m:0.051)
SUCLG2 (r2m:0.05)
$\log_2$CPM
Gestational Age (weeks)
12
20
28
36
5.0
5.5
6.0
6.5
7.0
-2
0
2
4
6
4
5
6
7
8
2.5
3.0
3.5
4.0
4.5

Supplementary Figure 6.
Top 100 cfRNAs significantly decreasing (negative coefficient) by gestational age

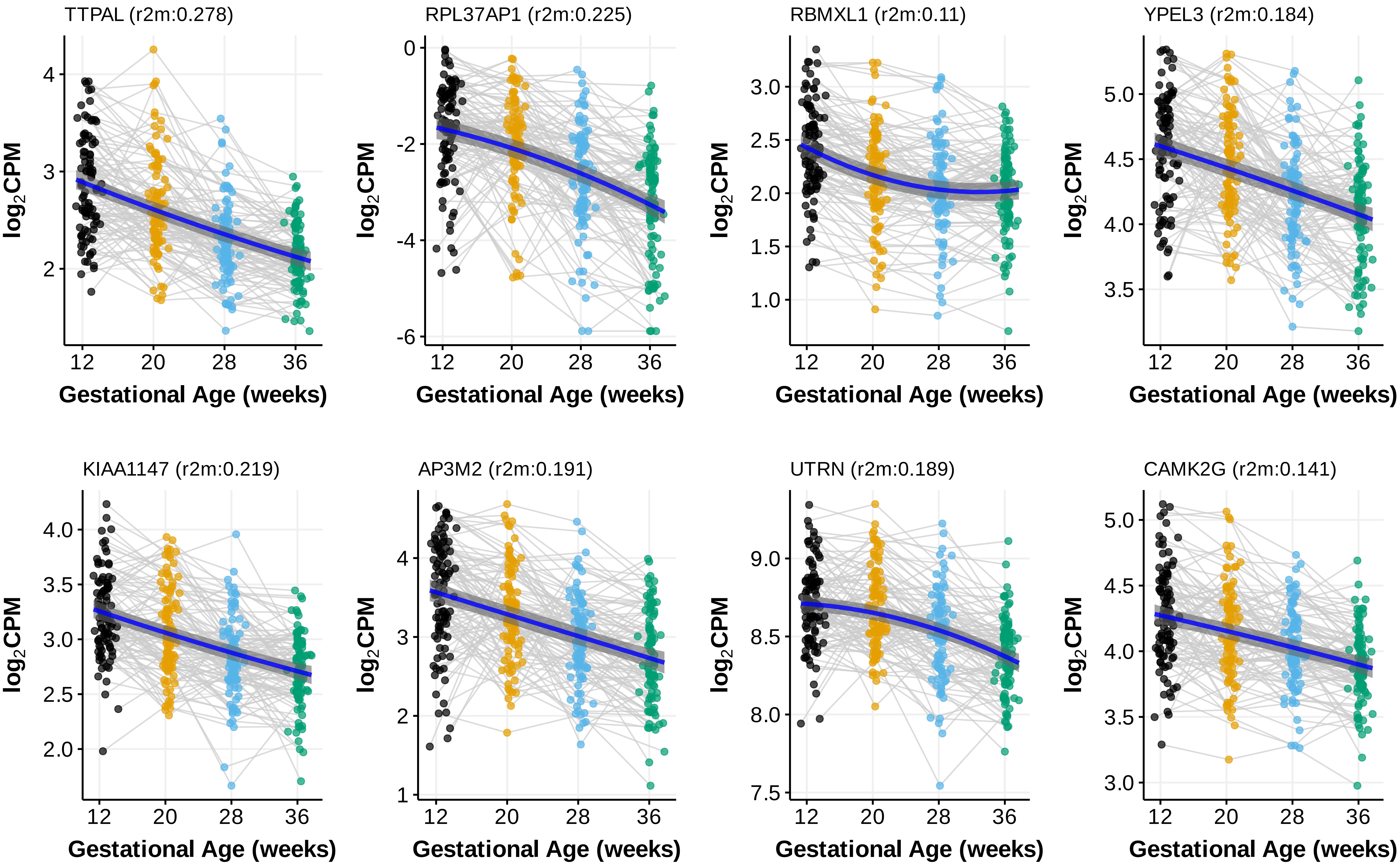

TTPAL (r2m:0.278)
RPL37AP1 (r2m:0.225)
RBMXL1 (r2m:0.11)
YPEL3 (r2m:0.184)
KIAA1147 (r2m:0.219)
AP3M2 (r2m:0.191)
UTRN (r2m:0.189)
CAMK2G (r2m:0.141)
log2CPM
Gestational Age (weeks)
12
20
28
36

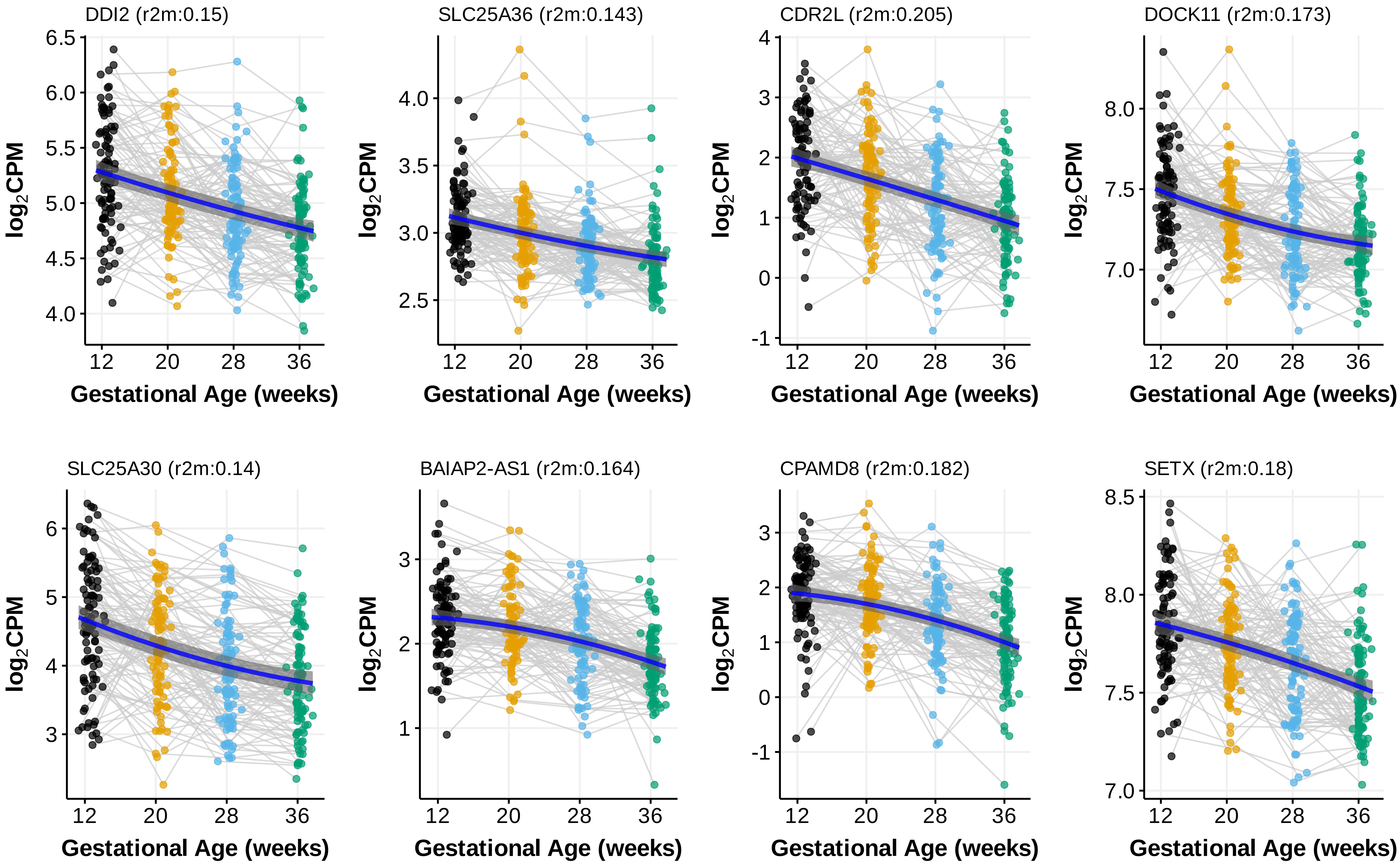
DDI2 (r2m:0.15)
SLC25A36 (r2m:0.143)
CDR2L (r2m:0.205)
DOCK11 (r2m:0.173)
SLC25A30 (r2m:0.14)
BAIAP2-AS1 (r2m:0.164)
CPAMD8 (r2m:0.182)
SETX (r2m:0.18)
log2CPM
Gestational Age (weeks)
12
20
28
36

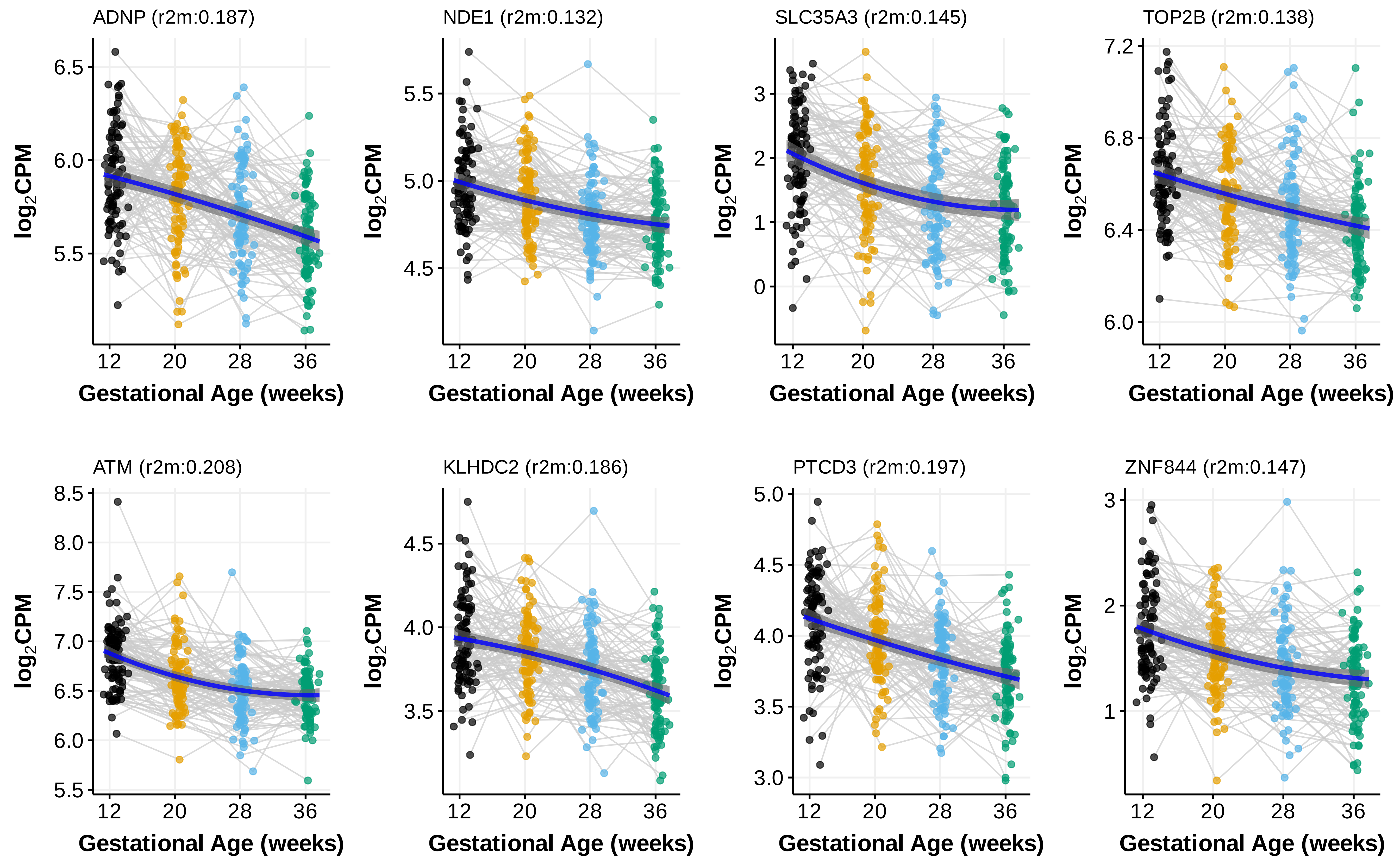
ADNP (r2m:0.187)
NDE1 (r2m:0.132)
SLC35A3 (r2m:0.145)
TOP2B (r2m:0.138)
ATM (r2m:0.208)
KLHDC2 (r2m:0.186)
PTCD3 (r2m:0.197)
ZNF844 (r2m:0.147)
log2CPM
Gestational Age (weeks)
12
20
28
36

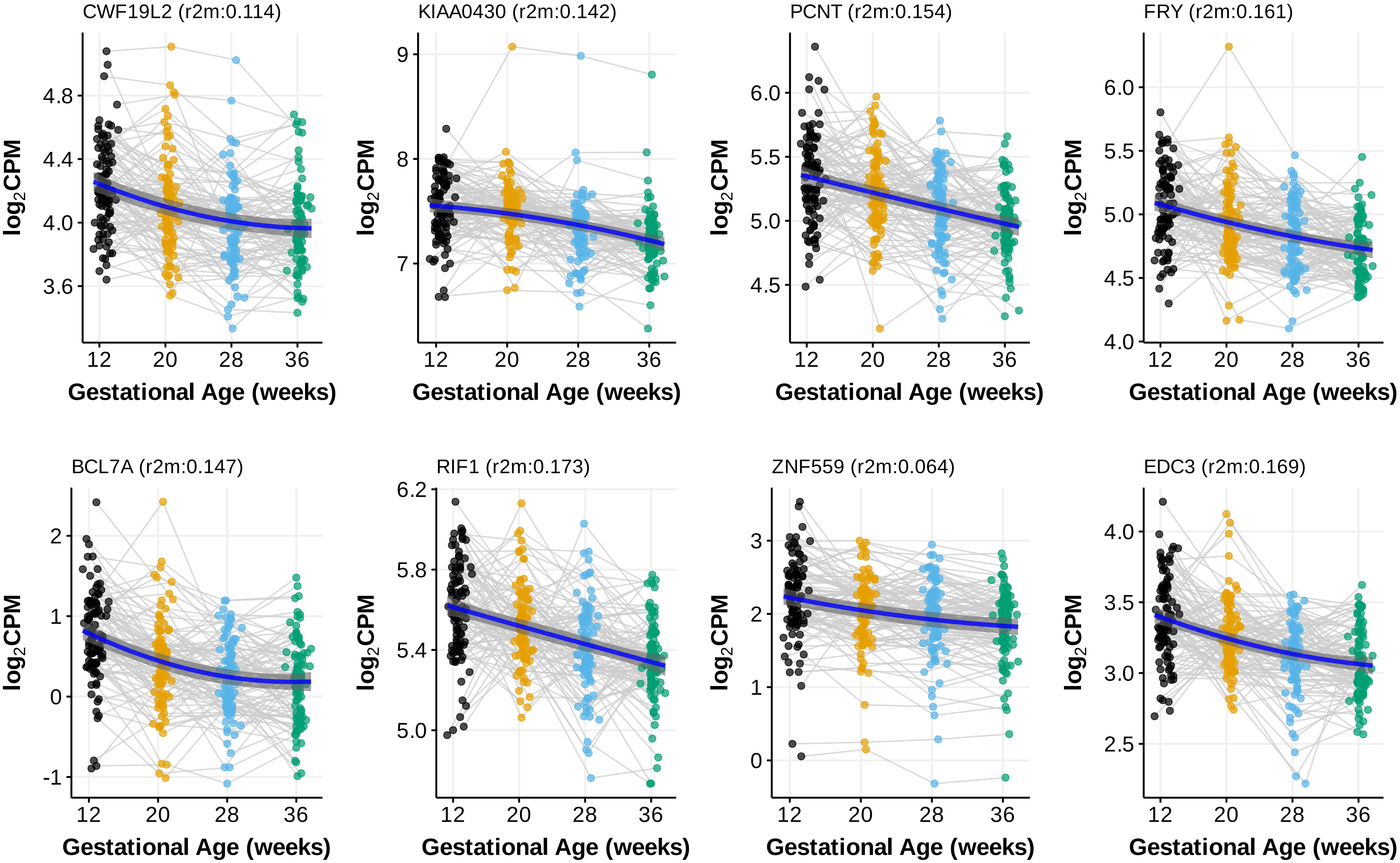

CWF19L2 (r2m:0.114)
KIAA0430 (r2m:0.142)
PCNT (r2m:0.154)
FRY (r2m:0.161)
BCL7A (r2m:0.147)
RIF1 (r2m:0.173)
ZNF559 (r2m:0.064)
EDC3 (r2m:0.169)
$\log_2$CPM
Gestational Age (weeks)
12
20
28
36

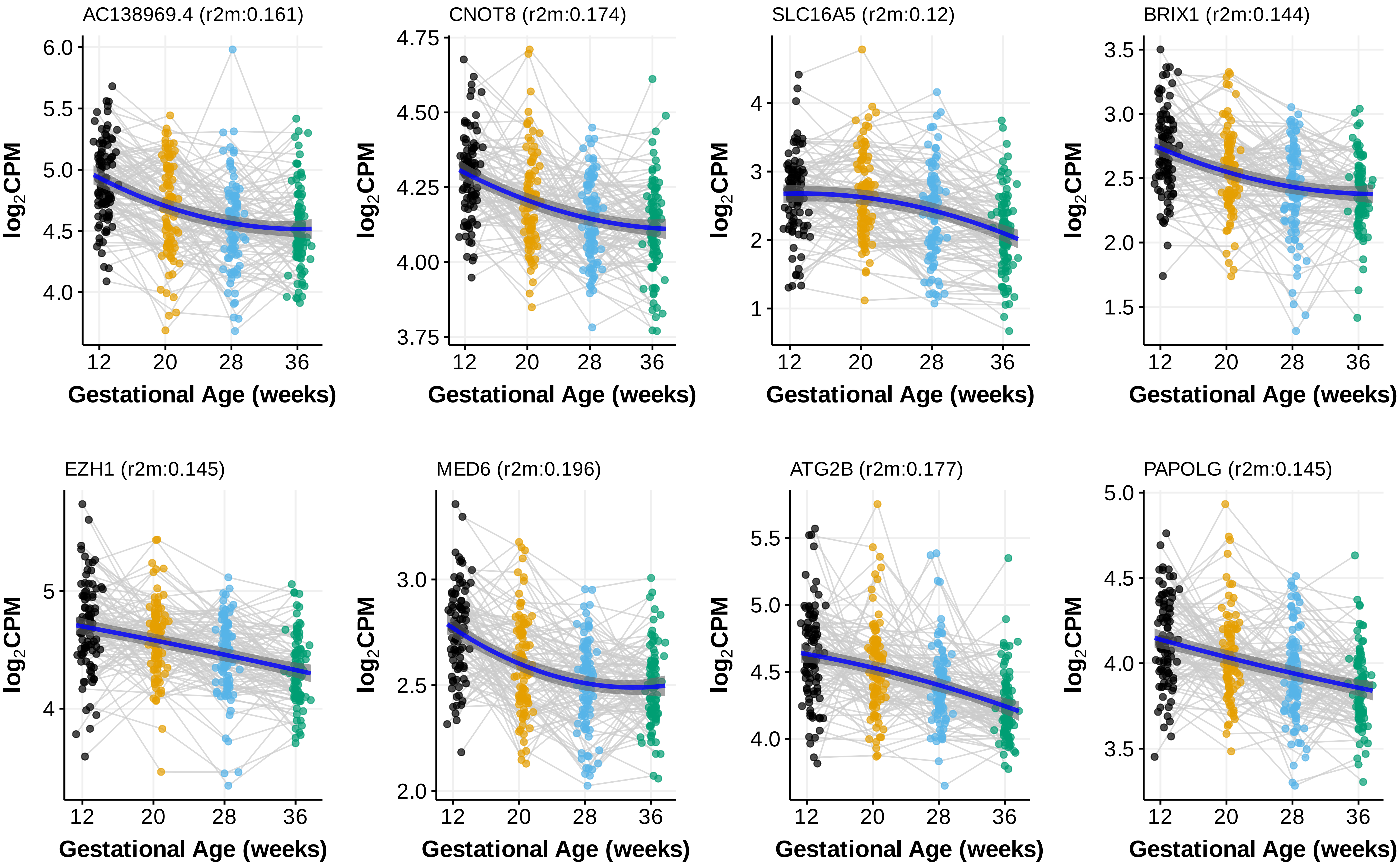
AC138969.4 (r2m:0.161)
CNOT8 (r2m:0.174)
SLC16A5 (r2m:0.12)
BRIX1 (r2m:0.144)
EZH1 (r2m:0.145)
MED6 (r2m:0.196)
ATG2B (r2m:0.177)
PAPOLG (r2m:0.145)
log_2CPM
Gestational Age (weeks)
12
20
28
36

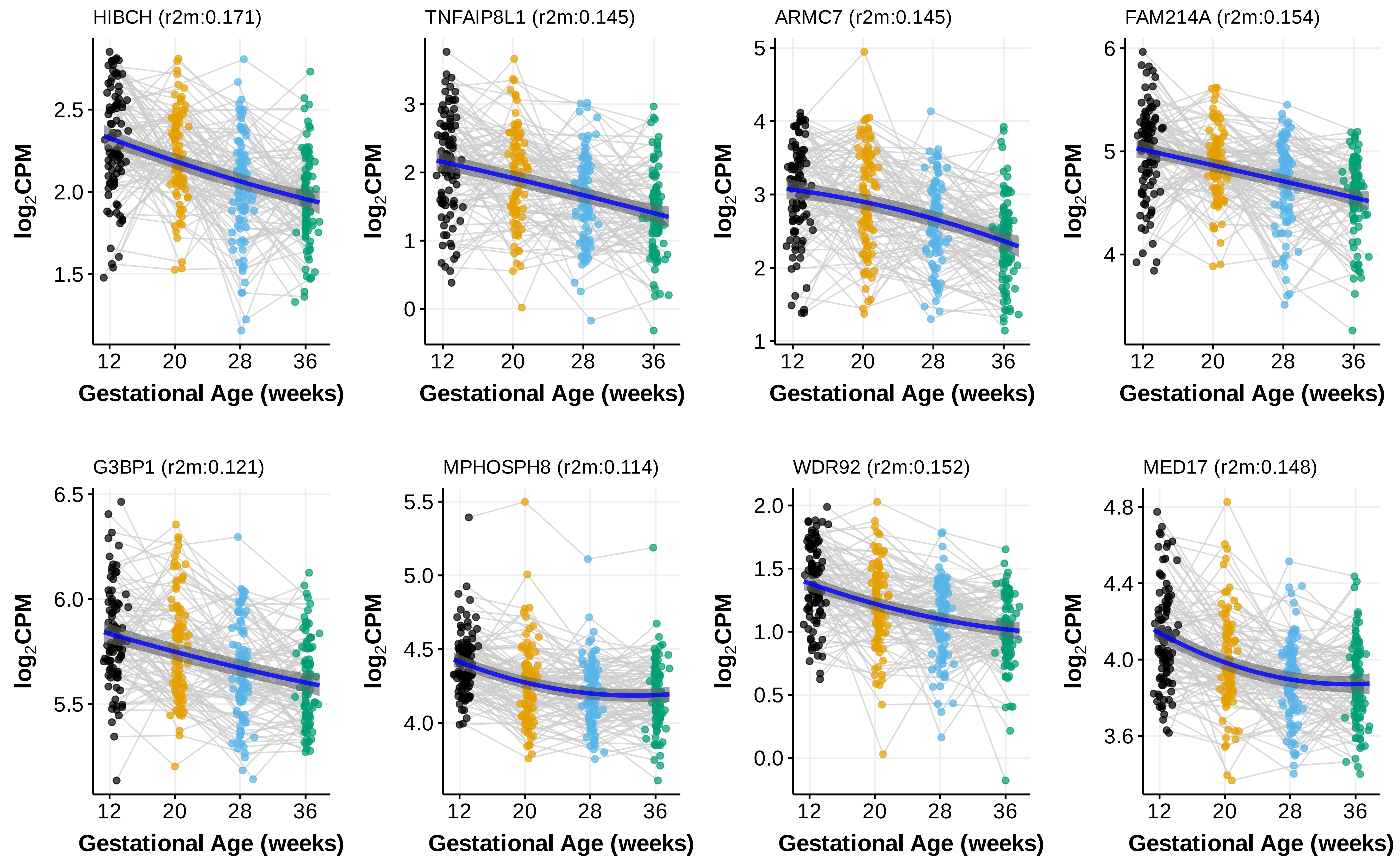

HIBCH (r2m:0.171)
TNFAIP8L1 (r2m:0.145)
ARMC7 (r2m:0.145)
FAM214A (r2m:0.154)
G3BP1 (r2m:0.121)
MPHOSPH8 (r2m:0.114)
WDR92 (r2m:0.152)
MED17 (r2m:0.148)
log2CPM
Gestational Age (weeks)
12
20
28
36

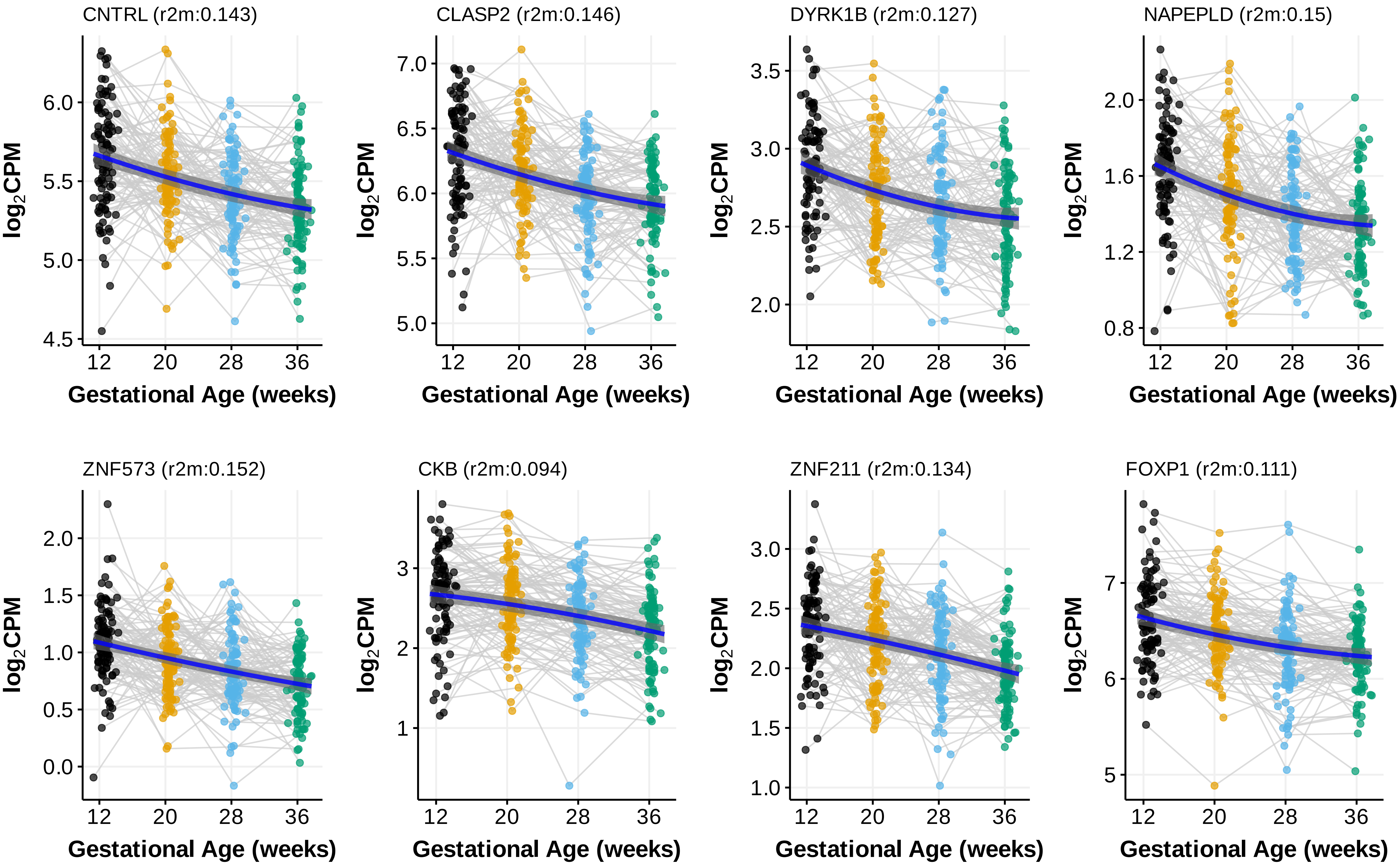

CNTRL (r2m:0.143)
CLASP2 (r2m:0.146)
DYRK1B (r2m:0.127)
NAPEPLD (r2m:0.15)
ZNF573 (r2m:0.152)
CKB (r2m:0.094)
ZNF211 (r2m:0.134)
FOXP1 (r2m:0.111)
log_2CPM
Gestational Age (weeks)
12
20
28
36

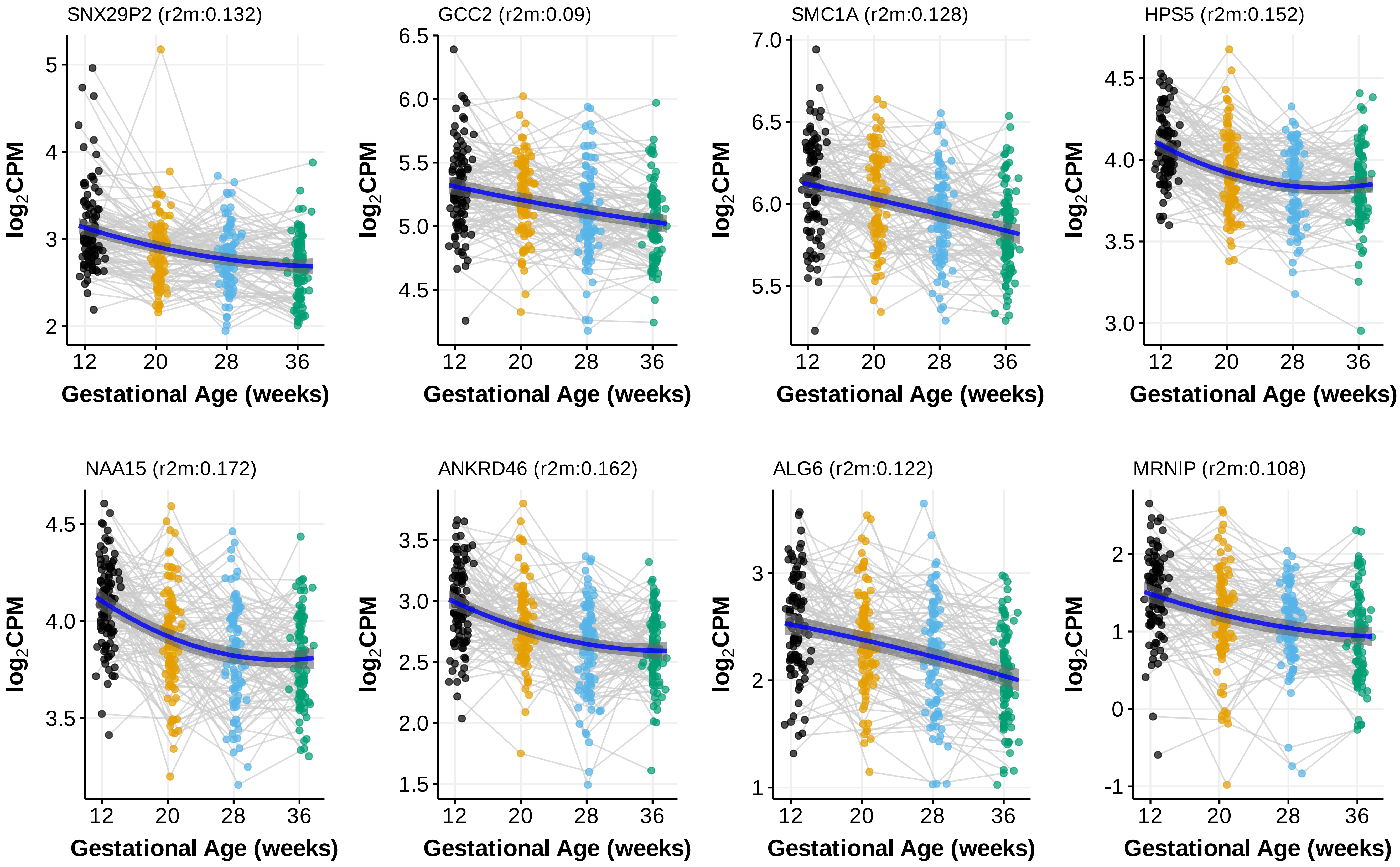

SNX29P2 (r2m:0.132)
GCC2 (r2m:0.09)
SMC1A (r2m:0.128)
HPS5 (r2m:0.152)
NAA15 (r2m:0.172)
ANKRD46 (r2m:0.162)
ALG6 (r2m:0.122)
MRNIP (r2m:0.108)
$\log_2$CPM
Gestational Age (weeks)
12
20
28
36

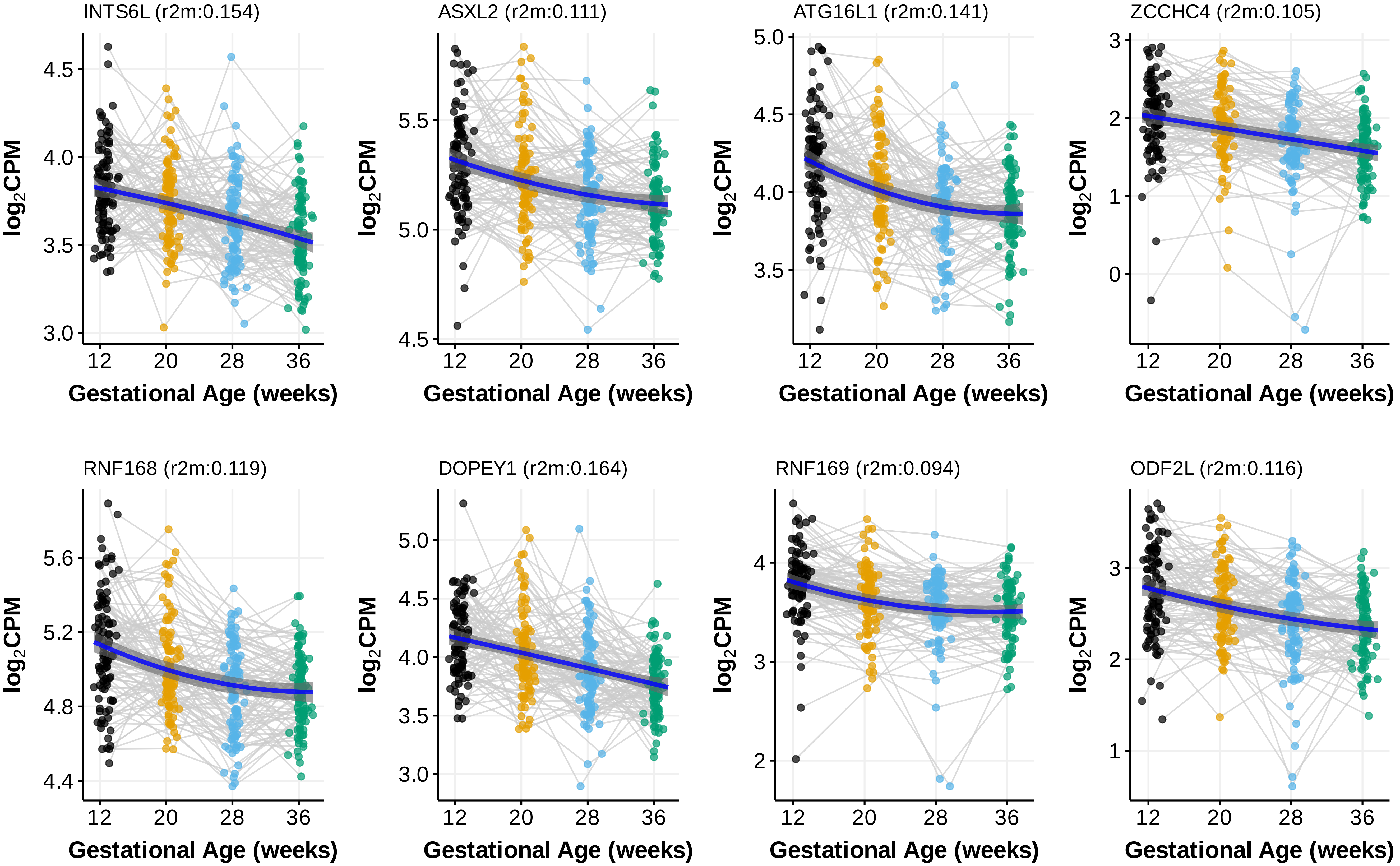
INTS6L (r2m:0.154)
ASXL2 (r2m:0.111)
ATG16L1 (r2m:0.141)
ZCCHC4 (r2m:0.105)
RNF168 (r2m:0.119)
DOPEY1 (r2m:0.164)
RNF169 (r2m:0.094)
ODF2L (r2m:0.116)
log2CPM
Gestational Age (weeks)
12
20
28
36

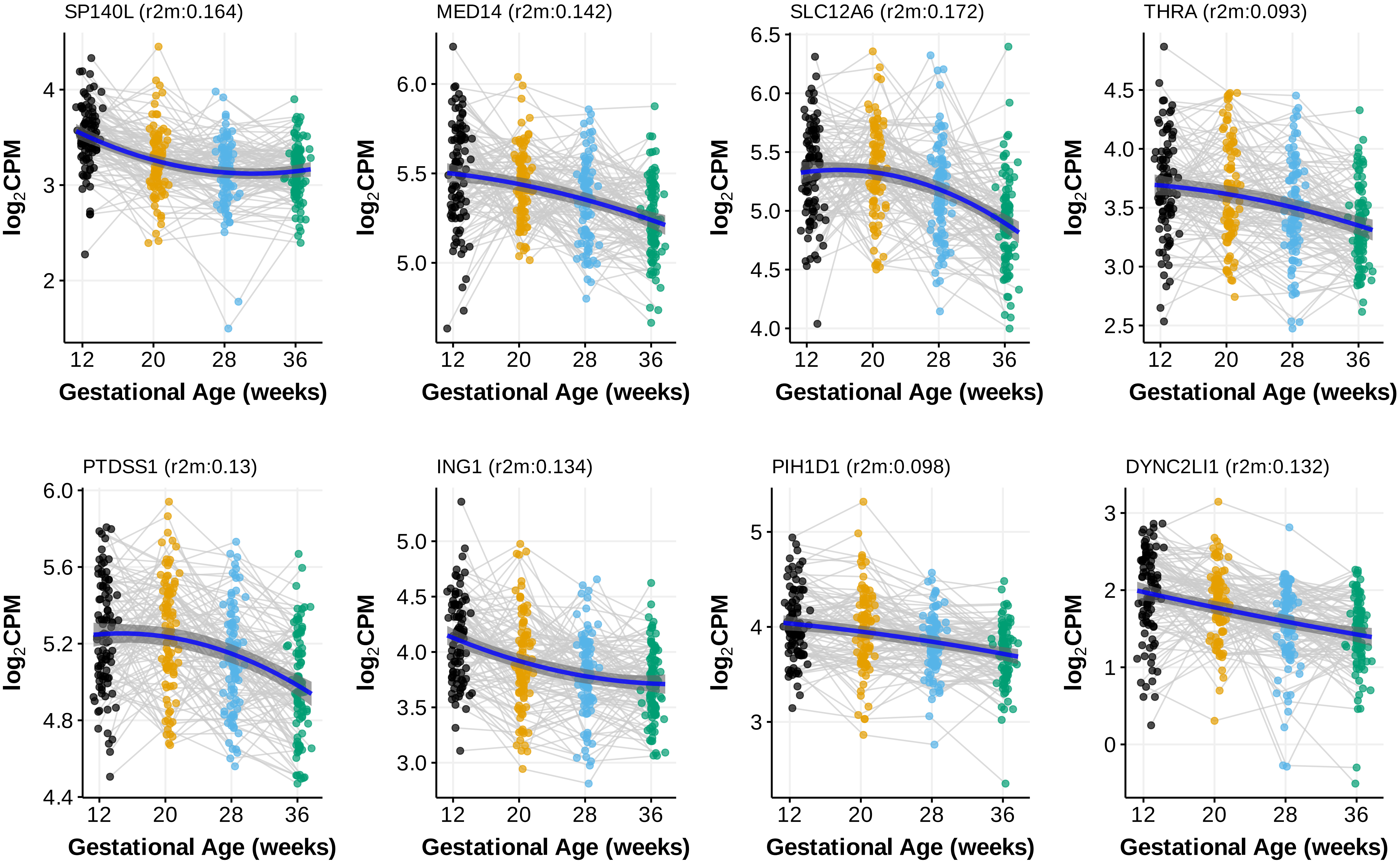
SP140L (r2m:0.164)
MED14 (r2m:0.142)
SLC12A6 (r2m:0.172)
THRA (r2m:0.093)
PTDSS1 (r2m:0.13)
ING1 (r2m:0.134)
PIH1D1 (r2m:0.098)
DYNC2LI1 (r2m:0.132)
log2CPM
Gestational Age (weeks)
12
20
28
36

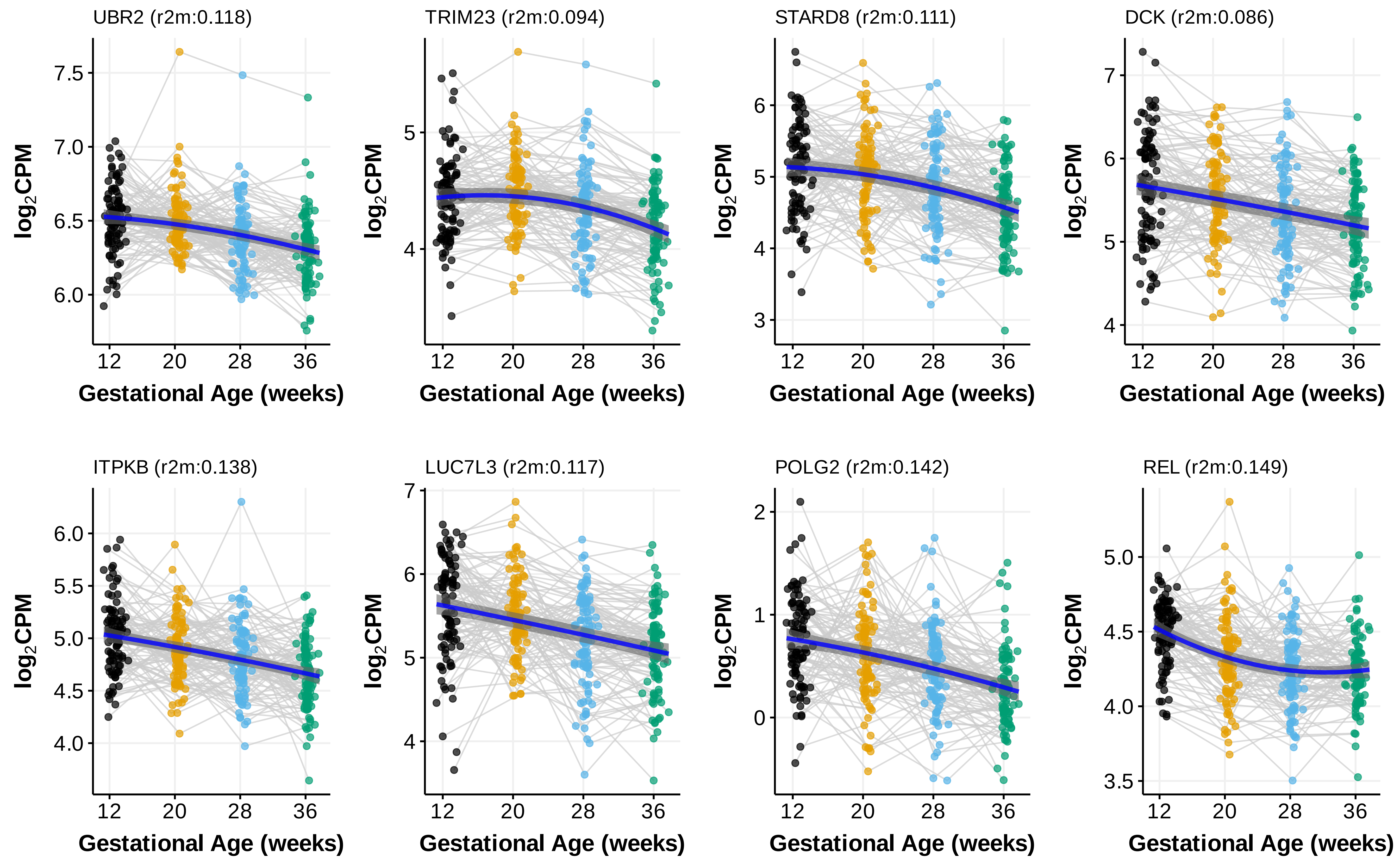
UBR2 (r2m:0.118)
TRIM23 (r2m:0.094)
STARD8 (r2m:0.111)
DCK (r2m:0.086)
ITPKB (r2m:0.138)
LUC7L3 (r2m:0.117)
POLG2 (r2m:0.142)
REL (r2m:0.149)
log_2CPM
Gestational Age (weeks)
12
20
28
36

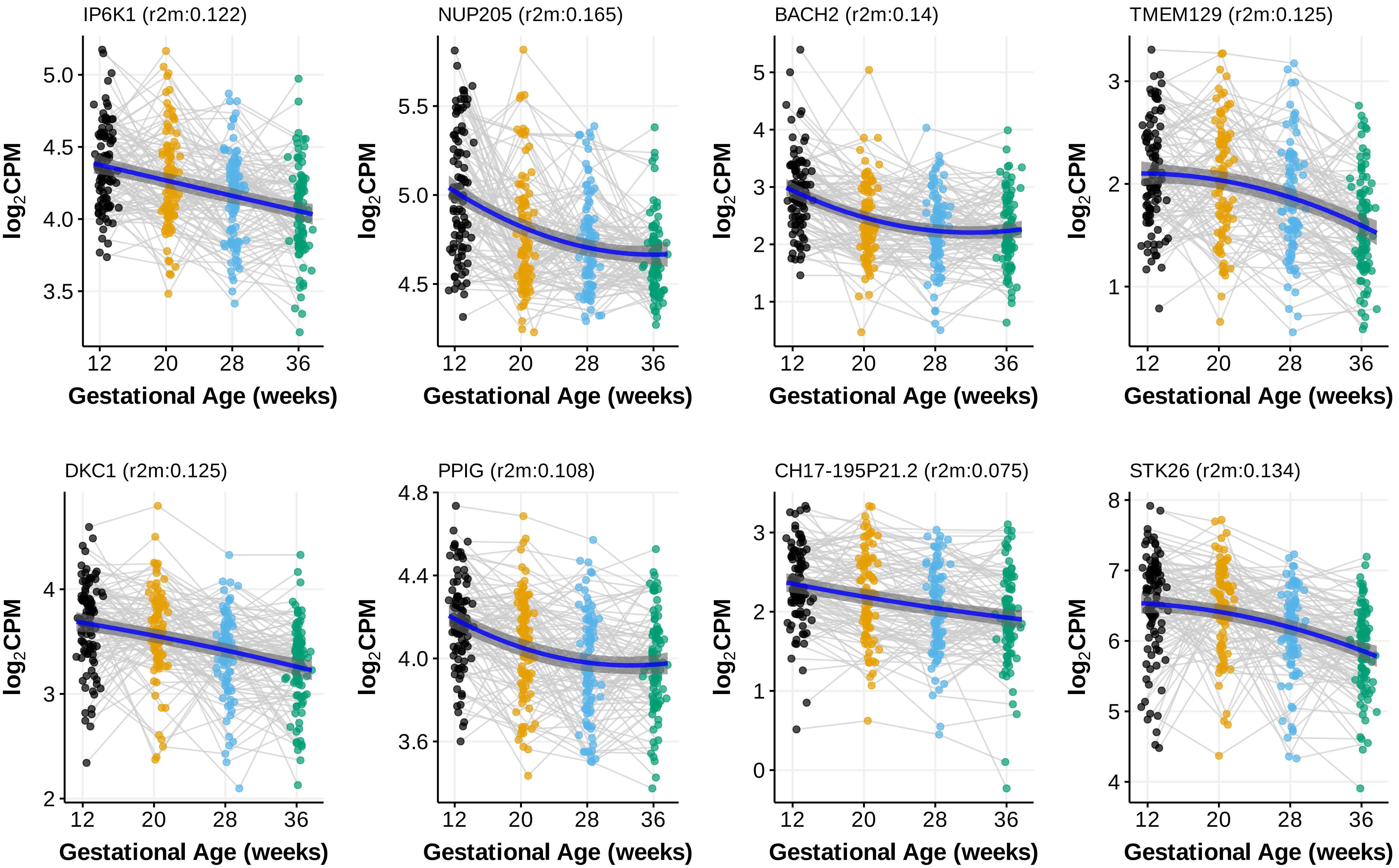

IP6K1 (r2m:0.122)
NUP205 (r2m:0.165)
BACH2 (r2m:0.14)
TMEM129 (r2m:0.125)
DKC1 (r2m:0.125)
PPIG (r2m:0.108)
CH17-195P21.2 (r2m:0.075)
STK26 (r2m:0.134)
log2CPM
Gestational Age (weeks)
12
20
28
36

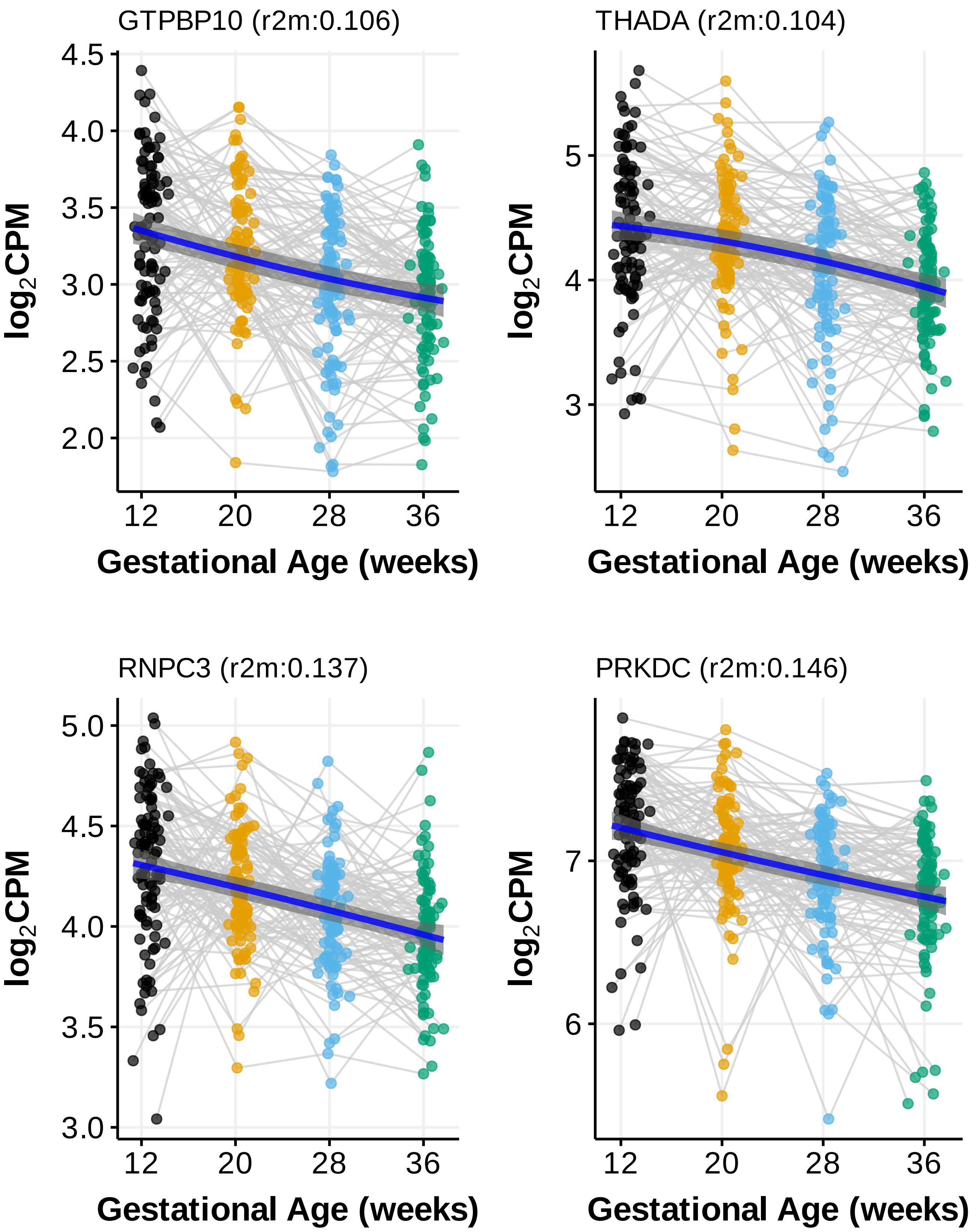

GTPBP10 (r2m:0.106)
THADA (r2m:0.104)
RNPC3 (r2m:0.137)
PRKDC (r2m:0.146)
log2CPM
Gestational Age (weeks)
12
20
28
36
