# Supplementary material for: Raised Leptin and Pappalysin2 cell-free RNAs are the hallmarks of pregnancies complicated by preeclampsia with fetal growth restriction
Source: Nat Commun. 2025 Jul 18;16:6614. doi: 10.1038/s41467-025-61931-7 (PMC12274584; doi:10.1038/s41467-025-61931-7)
Supplement: Supplementary file 3 — Description of Additional Supplementary Files [file 41467_2025_61931_MOESM3_ESM.pdf]

Name	Description
Supplementary data 1	Descriptive statistic of sample matching
Supplementary data 2	8054 differentially expressed genes from logistic regression analysis
Supplementary data 3	5898 differentially expressed genes from edgeR
Supplementary data 4	1445 differentially expressed genes from DESeq2
Supplementary data 5	The 345 differentially expressed genes
Supplementary data 6	The predictive performance and the list of selected cfRNAs during 5-fold CV with 5 repetitions
Supplementary data 7	The summary of 5-fold CV with 5 repetitions
Supplementary data 8	The final training models from Elastic net
Supplementary data 9	A like-for-like comparison of the predictive performance between the original model and the combined model
Supplementary data 10	The 129 differentially expressed genes by gestational age group
Supplementary data 11	Conditional and marginal coefficient of determination of mixed-effect models with a linear and quadratic term
Supplementary data 12	Conditional and marginal coefficient of determination of mixed-effect models with a linear term only
Supplementary data 13	Quality control statistics of RNA-seq data

1
