# Supplementary material for: Raised Leptin and Pappalysin2 cell-free RNAs are the hallmarks of pregnancies complicated by preeclampsia with fetal growth restriction
Source: Nat Commun. 2025 Jul 18;16:6614. doi: 10.1038/s41467-025-61931-7 (PMC12274584; doi:10.1038/s41467-025-61931-7)
Supplement: Supplementary file 5 — Reporting Summary [file 41467_2025_61931_MOESM5_ESM.pdf]

nature portfolio

Corresponding author(s): D. S. Charnock-Jones

Last updated by author(s): 19th June 2025

Reporting Summary

Nature Portfolio wishes to improve the reproducibility of the work that we publish. This form provides structure for consistency and transparency in reporting. For further information on Nature Portfolio policies, see our Editorial Policies and the Editorial Policy Checklist.

Statistics

For all statistical analyses, confirm that the following items are present in the figure legend, table legend, main text, or Methods section.

n/a	Confirmed	
☐	☒	The exact sample size (*n*) for each experimental group/condition, given as a discrete number and unit of measurement
☐	☒	A statement on whether measurements were taken from distinct samples or whether the same sample was measured repeatedly
☐	☒	The statistical test(s) used AND whether they are one- or two-sided<br>*Only common tests should be described solely by name; describe more complex techniques in the Methods section.*
☒	☐	A description of all covariates tested
☐	☒	A description of any assumptions or corrections, such as tests of normality and adjustment for multiple comparisons
☐	☒	A full description of the statistical parameters including central tendency (e.g. means) or other basic estimates (e.g. regression coefficient) AND variation (e.g. standard deviation) or associated estimates of uncertainty (e.g. confidence intervals)
☐	☒	For null hypothesis testing, the test statistic (e.g. *F*, *t*, *r*) with confidence intervals, effect sizes, degrees of freedom and *P* value noted<br>*Give P values as exact values whenever suitable.*
☒	☐	For Bayesian analysis, information on the choice of priors and Markov chain Monte Carlo settings
☒	☐	For hierarchical and complex designs, identification of the appropriate level for tests and full reporting of outcomes
☐	☒	Estimates of effect sizes (e.g. Cohen's *d*, Pearson's *r*), indicating how they were calculated

Our web collection on statistics for biologists contains articles on many of the points above.

Software and code

Policy information about availability of computer code

Data collection	The only software used to collect data was the standard NovaSeq 6000 (Illumina) sequencing machine software.
Data analysis	DRAGEN (Dynamic Read Analysis for GENomics) RNA pipeline (v3.8), Salmon (v1.5.2), StringTie (v2.2.1), Ensembl (v88), tximeta (v1.8.5), DESeq2 (v1.30.0), edgeR (v3.32.1), pROC (v1.17.0.1), ggvenn (v0.1.0), glParallel (https://gitlab.developers.cam.ac.uk/ssg29/glparallel), caret (v6.0.94), glmnet (v.4.1.2), readxl (v1.3.1), data.table (v1.13.6), lme4 (v1.1.37), lmerTest (v3.1.3), MuMIn" (v1.48.4).

For manuscripts utilizing custom algorithms or software that are central to the research but not yet described in published literature, software must be made available to editors and reviewers. We strongly encourage code deposition in a community repository (e.g. GitHub). See the Nature Portfolio guidelines for submitting code & software for further information.

Data

Policy information about availability of data

All manuscripts must include a data availability statement. This statement should provide the following information, where applicable:

- Accession codes, unique identifiers, or web links for publicly available datasets
- A description of any restrictions on data availability
- For clinical datasets or third party data, please ensure that the statement adheres to our policy

The maternal plasma RNA-seq data have been deposited in the European Genome-phenome Archive (EGA, https://ega-archive.org/) with the following accession number: EGAD00001015416. All original code is publicly available from https://obsgynaecam.github.io/cell-free-rna-2024/

Research involving human participants, their data, or biological material

Policy information about studies with human participants or human data. See also policy information about sex, gender (identity/presentation), and sexual orientation and race, ethnicity and racism.

Reporting on sex and gender	Pregnant women were recruited to the POPS cohort. Control subjects were matched with controls based on fetal sex.
Reporting on race, ethnicity, or other socially relevant groupings	No analyses were performed which used ancestry as a variable.
Population characteristics	The POP study population was an unselected cohort of nulliparous women with a singleton pregnancy who delivered in Cambridge, UK. The cohort has been described in detail in our previous papers (Sovio et al, Lancet 2015;386 (10008):2089-2097;Gaccioli et al, Placenta 2017;59(Supp1):S17-S25; Gong et al, JCI Insight 2018;3(13):e120723).
Recruitment	See above
Ethics oversight	Ethical approval for the study was given by the Cambridgeshire 2 Research Ethics Committee (reference number 07/ H0308/163) of the NHS Health Research Authority and all participants provided written informed consent

Note that full information on the approval of the study protocol must also be provided in the manuscript.

Field-specific reporting

Please select the one below that is the best fit for your research. If you are not sure, read the appropriate sections before making your selection.

☒ Life sciences ☐ Behavioural & social sciences ☐ Ecological, evolutionary & environmental sciences

For a reference copy of the document with all sections, see nature.com/documents/nr-reporting-summary-flat.pdf

Life sciences study design

All studies must disclose on these points even when the disclosure is negative.

Sample size	All cases of PE with FGR available in the POPS cohort were used. These were matched with controls at a 1:4 ratio.
Data exclusions	From among eligible non-cases, we excluded women who had missing data on body mass index (BMI) at 12wkGA, smoking at 12wkGA or fetal sex, women who lacked one or more samples at 12-36wkGA or sampling was performed outside the specified acceptable GA range.
Replication	The predictive model was developed in the preterm cases and validated using the term cases. External validation was carried out using the publicly available data from Munchel et al 2020.
Randomization	Cases and controls were fully randomised for analysis
Blinding	Sample collection was blinded during the POP studiy. Researchers were blinded to the pregnancy outcome when performing the downstream experiments. Data were unblinded at the statistical analysis stage

Reporting for specific materials, systems and methods

We require information from authors about some types of materials, experimental systems and methods used in many studies. Here, indicate whether each material, system or method listed is relevant to your study. If you are not sure if a list item applies to your research, read the appropriate section before selecting a response.

Materials & experimental systems

n/a	Involved in the study	
☒	☐	Antibodies
☒	☐	Eukaryotic cell lines
☒	☐	Palaeontology and archaeology
☒	☐	Animals and other organisms
☒	☐	Clinical data
☒	☐	Dual use research of concern
☒	☐	Plants

Methods

n/a	Involved in the study	
☒	☐	ChIP-seq
☒	☐	Flow cytometry
☒	☐	MRI-based neuroimaging

Plants

Seed stocks	*Report on the source of all seed stocks or other plant material used. If applicable, state the seed stock centre and catalogue number. If plant specimens were collected from the field, describe the collection location, date and sampling procedures.*
Novel plant genotypes	*Describe the methods by which all novel plant genotypes were produced. This includes those generated by transgenic approaches, gene editing, chemical/radiation-based mutagenesis and hybridization. For transgenic lines, describe the transformation method, the number of independent lines analyzed and the generation upon which experiments were performed. For gene-edited lines, describe the editor used, the endogenous sequence targeted for editing, the targeting guide RNA sequence (if applicable) and how the editor was applied.*
Authentication	*Describe any authentication procedures for each seed stock used or novel genotype generated. Describe any experiments used to assess the effect of a mutation and, where applicable, how potential secondary effects (e.g. second site T-DNA insertions, mosiacism, off-target gene editing) were examined.*
